# Coelectrolysis of PET and CO_2_ Using an Electrochemically Restructured Co-MOF-74 Anode and a Polymeric Co-Phthalocyanine Cathode

**DOI:** 10.1021/acsami.5c22269

**Published:** 2026-01-20

**Authors:** Raúl Rojas-Luna, Lewis S. Cousins, Rhiannon Germaney, Dolores G. Gil-Gavilán, Miguel Castillo-Rodríguez, Dora-Alicia Garcia Osorio, Thomas Doughty, Dolores Esquivel, Charles E. Creissen, Souvik Roy

**Affiliations:** † Department of Chemistry, School of Natural Sciences, 4547 University of Lincoln, Green Lane, Lincoln LN6 7DL, U.K.; ‡ School of Chemical and Physical Sciences, 4212 Keele University, Staffordshire ST5 5BG, U.K.; § Departamento de Química Orgánica, Instituto Químico para la Energía y el Medioambiente (IQUEMA), Facultad de Ciencias, 16735 Universidad de Córdoba, Campus de Rabanales, Edificio Marie Curie, 14071 Córdoba, Spain; ∥ Departamento de Física Aplicada, Radiología y Medicina Física, Universidad de Córdoba, Campus de Rabanales, 14071 Córdoba, Spain; ⊥ Department of Chemistry, School of Natural Sciences, University of Lincoln, Green Lane, Lincoln LN6 7DL, U.K.

**Keywords:** CO_2_ electrolyzer, metal−organic framework, electrochemical restructuring, ethylene glycol oxidation, flow-cell electrolyzer, plastic upcycling

## Abstract

Mitigating carbon emissions and plastic waste is a pressing societal challenge due to the disruptive environmental impact of incremental accumulation. A promising strategy to address both issues is coelectrolysis of CO_2_ and PET-plastic waste to high-value commodity chemicals. Here, we report electrocatalytic upcycling of polyethylene terephthalate (PET) plastic to formate and terephthalic acid using a cobalt-based metal–organic framework (Co-MOF-74). The electrocatalyst underwent oxidative restructuring to cobalt oxyhydroxide under operating conditions and exhibited near-unity faradaic efficiency (FE) for the ethylene glycol oxidation reaction (EGOR) to formate during short-term electrolysis. Notably, EGOR required 0.23 V lower potential compared to the conventional oxygen evolution reaction (OER) at a current density of 100 mA cm^–2^. When coupled with a CO_2_ reducing cathode, a maximum combined FE of 156% was achieved for formate (anode) and syngas (cathode) at a cell voltage (*E*_cell_) of 1.6 V. Upon integration of the EGOR electrode in a CO_2_-fed flow cell, the coupled system required an *E*_cell_ of ∼2.3 V to operate at 75 mA cm^–2^. This work presents a promising integrated approach that offers a compelling solution for mitigating environmental pollution by enabling the electrochemical reforming of CO_2_ and plastic waste into valuable chemicals under cost-effective and energy-efficient conditions.

## Graphical abstract

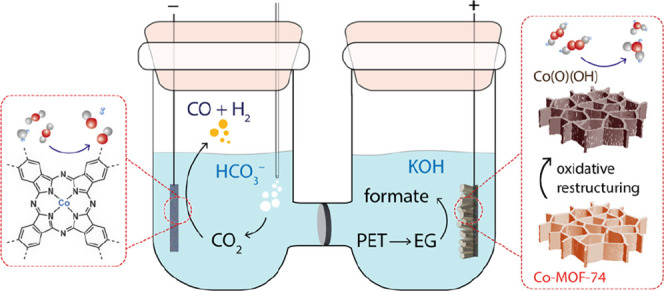

## Introduction

Electrocatalysis has emerged as a viable option for the sustainable production of chemicals and fuels.^1^ Powered by renewable electricity, abundant building blocks and waste materials can be transformed into value-added products that can feed into future circular chemical industries.^2,3^ In this context, CO_2_ electrolysis has emerged as a promising technology that can mitigate emissions while producing high-value carbon-based chemicals.^3–5^ Conventionally, CO_2_ reduction is coupled with the oxygen evolution reaction (OER) at the anode, which is considered to be a bottleneck due to its high overpotential requirement and sluggish reaction kinetics. This has spurred research into alternative electrooxidation reactions with more favorable thermodynamics and therefore the ability to reduce the electricity input and consequently costs for electrolysis.^6–8^ To that goal, polyethylene terephthalate (PET) plastic waste presents a largely untapped resource that can be electrochemically reformed to higher-value chemicals.^9^ Due to its robustness and excellent performance, PET is widely used in many fields, such as packaging, automotives, and textiles. However, accumulation of PET poses a serious environmental challenge due to its resistance toward natural degradation,^10,11^ leading to formation of microplastics that interferes with ecological systems. The polyester nature of the polymer chain makes it susceptible toward alkaline hydrolysis to terephthalate and ethylene glycol (EG) monomers (Scheme 1a). Electrochemical reforming of this PET hydrolysate offers a sustainable and potentially cost-effective alternative to traditional plastic recycling methods.^12^ Terephthalic acid (TPA) can be easily recovered from the hydrolysate by adjusting the pH, but recovery of EG is a demanding process due to its high boiling point (197 °C), viscosity (19.8 mPa s at 20 °C), and water miscibility.^13^ Electrooxidation of PET hydrolysate and its monomer EG to value-added products, such as glycolate or formate, presents a promising strategy for upcycling waste that can be paired with CO_2_ reduction to increase economic output while lowering energy input. Thermodynamically, technoeconomic analysis of paired coelectrolysis of CO_2_ and PET has indicated that the coupling strategy can potentially generate high net revenues.^14^ However, the design of the electrocatalyst at the anode still has much room for improvement in terms of product selectivity, Faradaic yield, and energy efficiency.

**Scheme 1:**
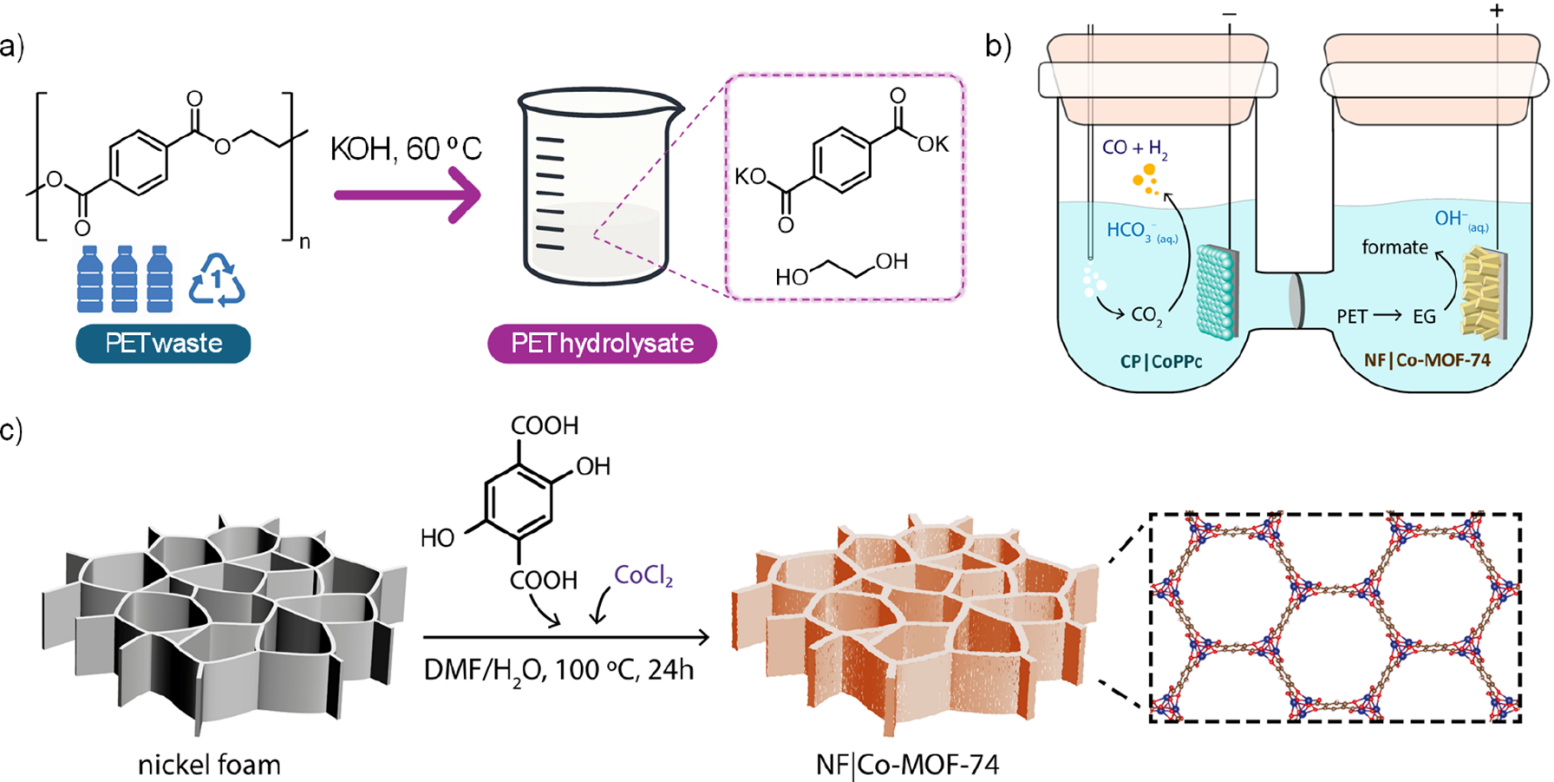
(a) Chemical Depolymerization of PET Waste via Alkaline Hydrolysis; (b) Schematic Illustration of the Coupled Electrolyzer, Featuring a NF|Co-MOF-74 Anode for EGOR and a CP|CoPPc Cathode for CO_2_R; (c) Schematic Illustration of the Synthesis of Co-MOF-74 on Nickel Foam (NF|Co-MOF-74)

The success of electrochemical upcycling in practical devices is largely reliant on the efficiency and intrinsic activity of the electrocatalyst toward EG oxidation reaction (EGOR). The commonly observed products for EGOR are formic acid (6 e^–^ process) and glycolic acid (4 e^–^ process), with the selectivity of the process depending on the mechanism of the electrooxidation pathway on the electrode surface.^15^ Overoxidation of the EGOR products to carbonate often lowers the Faradaic yields of the process, limiting its application in practical devices. An additional drawback is high overpotential requirement that can lead to competitive OER. In recent years, several non-noble metal-based electrocatalysts have been reported for EGOR displaying notable performance toward formate generation.^16–25^ For example, Zhou et al. reported a CoNi_0.25_P catalyst for conversion of PET hydrolysate to potassium diformate with >80% Faradaic efficiency (FE) at >100 mA cm^–2^ current density.^14^ Ma et al. recently demonstrated EGOR to formate with ∼90% FE using layered Ni–Co_9_S_8_ nanosheet arrays,^16^ while Li et al. achieved a comparable efficiency of 85% employing Co–Ni_2_P nanosheet arrays.^17^ In parallel, mixed metal oxides have also been developed as electrocatalysts for EGOR including CuO,^18^ CuCo_2_O_4_,^19^ NiCo_2_O_4_,^20^ CuCoO@rGO (rGO denotes reduced graphene oxide),^23^ Ni­(OH)_2_-decorated CuO electrode,^24^ and Mn/CoOOH.^25^ PET hydrolysate has also been efficiently converted to formate using metal hydroxide-based electrocatalysts, including oxygen-vacancy-rich Ni­(OH)_2_,^26^ and a Ni-MOF@MnCo–OH heterostructure.^27^ Very recently, layered double hydroxides (LDHs) have emerged as promising electrocatalysts for EGOR conversion to formate.^28–30^ The high activity and low cost for first-row transition-metal-based materials, such as metal oxides and hydroxides, have spurred interest in these materials. The high activity and low cost for first-row transition-metal-based materials, such as metal oxides and hydroxides, demonstrate that these materials are effective catalysts; however, methods to improve performance through modulation of structural properties and surface functionalities are required to further improve their performance.

Organic/inorganic hybrid materials such as metal–organic frameworks (MOFs) and their derivatives are an emerging class of microporous materials that are increasingly being applied as electrocatalysts for a range of reactions, such as hydrogen evolution reaction (HER),^31,32^ water oxidation reaction (WOR),^33–35^ and CO_2_ reduction (CO_2_R).^36^ Their modular structure offers an inherent advantage for catalyst design and performance optimization through changing the organic linker or modifying the structure of the node. While the MOFs have gained popularity in electrocatalytic applications, answers to questions regarding the structure of the active species have mostly remained elusive,^37^ especially when the electrocatalysts are subjected to harsh reaction conditions, such as strong alkaline electrolytes and highly oxidizing or reducing conditions. The coordination bonds between the organic linkers and the metal nodes are susceptible toward hydrolysis under alkaline conditions, often leading to irreversible restructuring of the MOF to form metal (oxy)­hydroxides,^38–40^ which is advantageous since the resulting MOOH species typically exhibit higher intrinsic activity, improved electronic conductivity, and more accessible active sites compared to the parent MOF. This structural reconstruction of MOFs has been exploited to design electrocatalysts with high activity toward OER,^41^ but their utilization toward electrooxidation of small organic molecules has rarely been investigated.^42^

In this work, we report electrocatalytic EGOR mediated by Co-MOF-74 grown on nickel foam and demonstrate the in situ restructuring of the MOF catalyst under operating conditions using a range of ex situ and in situ characterization tools. X-ray absorption spectroscopy (XAS) and operando Raman spectroscopy investigations confirmed the restructuring process under operating conditions and evolution of cobalt (oxy)­hydroxide as the active catalyst. The restructured materials exhibited high activity toward oxidation of EG and PET hydrolysate (*E*_1/2_ ∼0.23 V below the OER) and generated formate with high selectivity (FE ∼100% after 30 min). The Co-MOF-74-based anode was coupled with a hybrid CO_2_R cathode, featuring a polymeric cobalt phthalocyanine (CoPPc) capable of reducing CO_2_ to CO or syngas (Scheme 1b). We demonstrate that coelectrolysis of CO_2_ and PET hydrolysate is effective in both static (H-cell) conditions and in a gas-fed flow cell for high-rate electrolysis.

## Results and Discussion

### Synthesis and Characterization

Co-MOF-74 was synthesized by coordinating Co^2+^ ions using 2,5-dihydroxyterephthalic acid as organic linkers under solvothermal conditions following a reported method (Scheme 1c). Successful synthesis of microcrystalline Co-MOF-74 was confirmed by powder X-ray diffraction (PXRD) which shows characteristic diffraction peaks at 6.6°, 11.6°, 16.6°, and 17.9° corresponding to (110), (300), (211), and (321) planes, respectively (Figure S1, CCDC number 270293).^43^ The structure of Co-MOF-74 consists of hexagonal 1D channels constructed by an edge-sharing CoO_5_ square pyramidal polyhedral. Scanning electron microscopy (SEM) images revealed hexagonal 5–15 μm long rod-shaped crystals, consistent with previous reports on Co-MOF-74 (Figures 1a and S2).^44^ The high-resolution XPS spectrum of the Co 2p region displayed two dominant peaks at ∼780.0 and ∼795.9 eV corresponding to 2p_3/2_ and 2p_1/2_ signals (Figure 1b). The broad satellite peaks are consistent with Co^2+^ paramagnetic centers. For electrochemical investigation, Co-MOF-74 was directly grown on nickel foam (NF) under solvothermal conditions, and its purity was confirmed by PXRD (Figure 1c). Uniform coverage of the electrodes was confirmed by SEM images (Figure 1a). Transmission electron microscopy (TEM) further corroborated a crystalline structure of the surface-grown Co-MOF74 film (Figure S3). The selected area electron diffraction (SAED) patterns exhibited individual spots with interplanar distances of 1.29, 0.73, and 0.43 nm, which can be indexed to (110), (300), and (401) crystallographic planes of the Co-MOF-74 lattice.^43,45^ Successful synthesis of Co-MOF-74 on the nickel foam surface was further confirmed by infrared and Raman spectroscopy (Figure 1d,e). ATR-IR of the surface grown material displayed characteristic bands at 1550 and 1404 cm^–1^ due to asymmetric and symmetric stretching of the carboxylate, respectively. The Raman spectrum of NF|Co-MOF-74 exhibits three prominent peaks at 817, 1560, and 1619 cm^–1^, originating from the C–H bending and C = C stretching vibration modes of the benzene ring in the organic linker (2,5-dihydroxyterephthalate). Two intense peaks at 1415 and 1275 cm^–1^ are attributed to ν­(COO^–^) and ν­(C–O) vibrations, while the 566 cm^–1^ band is assigned to Co–O bond vibration.^46^

**Figure 1:**
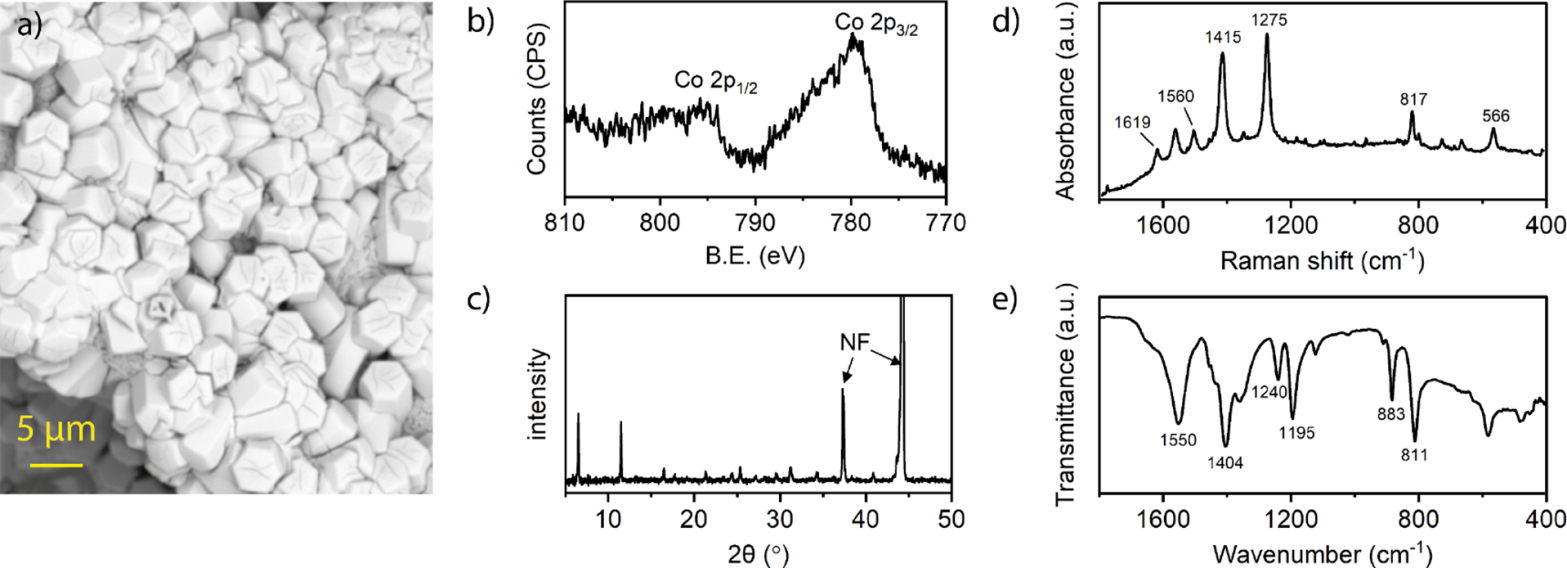
(a) SEM image of pristine NF|Co-MOF-74 (BSE detector). (b) Co 2p region of the XPS of Co-MOF-74. (c) Powder X-ray diffraction pattern of NF|Co-MOF-74. (d) Raman and (e) ATR-IR spectra of Co-MOF-74.

### Electrochemical Studies

In a three-electrode setup, a series of electrochemical analytical methods were used to investigate the electrocatalytic activity and selectivity of the Co-MOF-74 electrodes for the oxidation of ethylene glycol and PET hydrolysate in a KOH electrolyte. The catalyst-coated NF electrode (NF|Co-MOF-74) was conditioned by cycling between 0.8 and 1.8 V vs RHE in 1 M KOH. This showed a gradual change in the shape of the voltammogram with an increasing number of scans which became consistent after ∼50 scans (Figure 2a). The first scan exhibited two broad oxidation waves at ∼1.25 V and ∼1.49 V that coalesced into a single oxidation peak at ∼1.40 V over successive scans, suggesting structural changes within the material under oxidative conditions. The return scan showed an incremental increase in peak current of the reduction wave at ∼1.05 V, consistent with the exposure of more active sites after 50 scans. This quasi-reversible redox process can be tentatively attributed to the Co^3+/2+^ couple in the material (red trace, Figure 2a).^47,48^ ICP-OES analysis of the electrode showed a total cobalt loading of 29.7 μmol cm^–2^, while the surface coverage of electroactive cobalt centers (Γ_Co_) was estimated to be 7.14 ± 0.13 μmol cm^–2^ from the integration of the Co^3+/2+^ reductive wave. This corresponds to approximately 24% of the cobalt species on NF being electroactive. The CV showed the onset potential of water oxidation reaction on the conditioned electrode at >1.6 V, beyond the potential of the Co^2+/3+^ oxidation process. As shown in Figure 2b, linear sweep voltammetry with the conditioned electrode (NF|Co-MOF-74) displayed Co^2+/3+^ oxidation at ∼1.30 V and onset of water oxidation at 1.58 V for a 100 mA cm^–2^ current density. The electrochemically conditioned NF|Co-MOF-74 electrode displayed a visible darkening of the coating from red brown to an almost black color (Figure 2a, inset), suggesting electronic restructuring of Co-MOF-74 material. This was accompanied by a change of color of the electrolyte from colorless to pale red during electrochemical cycling. In situ UV–vis spectroelectrochemistry of the electrolyte showed appearance of a new absorbance band at ∼520 nm under oxidizing conditions (Figure S4). We speculate that it could be caused by leaching of the 2,5-dihydroxyterephthalic acid linker and cobalt ions in the electrolyte.

**Figure 2:**
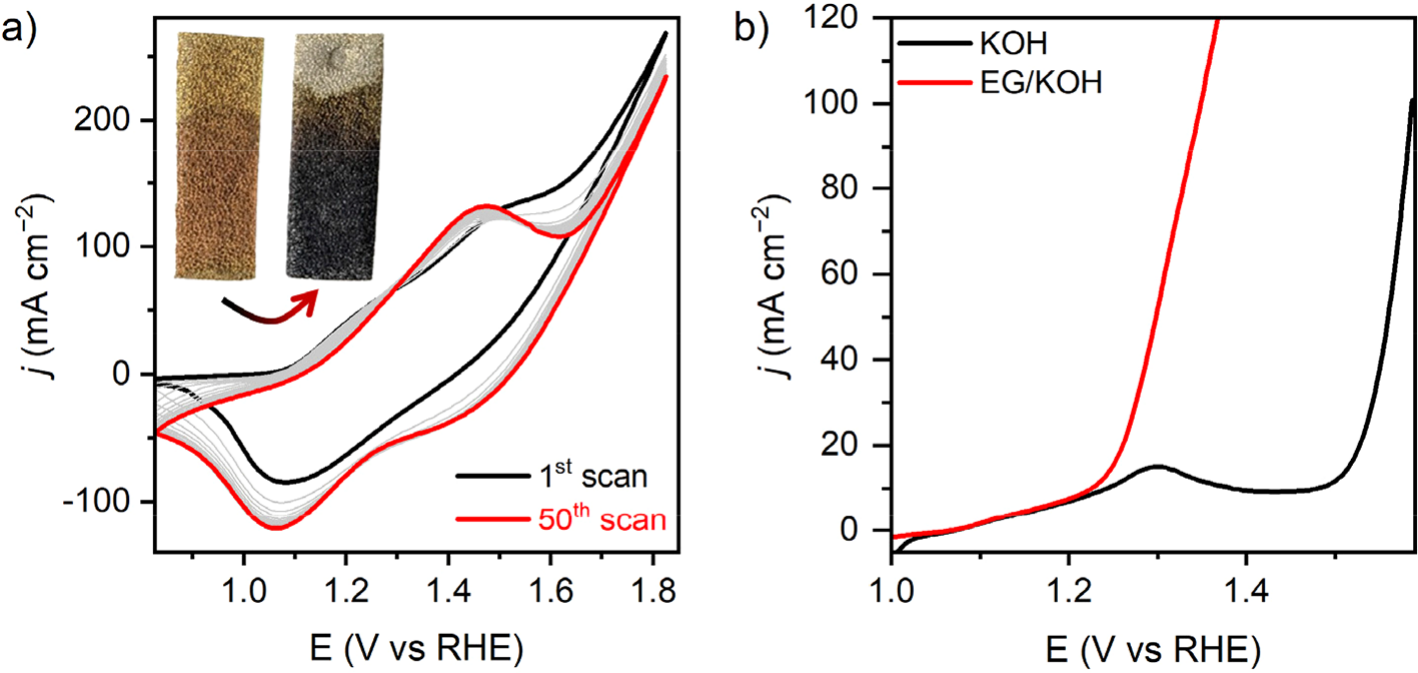
(a) Continuous cyclic voltammograms (activation process) of NF|Co-MOF-74 recorded in 1 M KOH at a scan rate of 20 mV s^–1^. The black and red traces show the 1st and 50th scans, respectively, and the intermediate scans are shown in gray. The inset shows photographs of as-synthesized NF|Co-MOF-74 (left) and conditioned NF|Co-MOF-74 (right). (b) Linear sweep voltammetry of NF|Co-MOF-74 recorded in 1 M KOH in the presence and absence of ethylene glycol (0.1 M) at a scan rate of 1 mV s^–1^ (90% *iR*-compensation).

In the presence of 0.1 M ethylene glycol (EG), a much earlier onset of catalytic current was observed, overlapping with the Co^2+/3+^ oxidation process and indicating activity toward ethylene glycol oxidation reaction (EGOR) (Figure 2b). To achieve a current density of 100 mA cm^–2^, a potential of only 1.35 V was required, indicating that the anodic potential for EGOR is 0.23 V lower than the OER in an alkaline medium. This performance is competitive with other benchmark electrocatalysts reported for EGOR under similar conditions (Table S2). Control experiments with a blank NF electrode showed a much higher onset potential for EGOR, confirming that the activity originates from the MOF-derived catalyst layer (Figure S5a). In comparison, pristine Co­(OH)_2_ electrodeposited on a NF electrode displayed significantly lower activity as an electrocatalyst for EGOR, with NF|Co­(OH)_2_ requiring 1.52 V to reach ∼100 mA cm^–2^ current density (Figure S5b), which highlights that the microstructure of MOF is crucial for achieving the high EGOR performance. To evaluate the intrinsic catalytic activity for EGOR of NF|Co-MOF-74, electrochemical impedance spectroscopy (EIS) tests were conducted, and the Tafel slopes were derived from the LSV data. As shown in Figure S6, the NF|Co-MOF-74 electrode exhibited a lower Tafel slope of 94 mv dec^–1^ for EGOR compared to OER (135 mV dec^–1^) and the Nyquist plot revealed lower charge transfer resistance (*R*_ct_) in the presence of EG in the electrolyte. These findings demonstrated the high intrinsic electrocatalytic activity of the material toward EGOR. EIS recorded at different potentials under EGOR conditions revealed that the charge transfer resistance decreased with increasing applied potential, which could be attributed to the efficient electron transfer during EG oxidation on the NF|Co-MOF-74 electrode (Figure S6c). The electrochemically active surface area (ECSA) was estimated by using the double-layer capacitance (*C*_dl_). Figure S7a–c display scan rate-dependent CVs recorded in the non-Faradaic region for the NF control electrode, the as-synthesized NF|Co-MOF-74, and the restructured NF|Co-MOF-74. The *C*_dl_ values, derived from the linear fit of capacitive current versus scan rate (Figure S7d), were determined as 0.64, 1.40, and 0.74 mF cm^–2^, respectively. Thus, a 2.2-fold increase in the number of catalytically active sites was observed upon the growth of Co-MOF-74 on the NF substrate. Nevertheless, the reconstructed NF|Co-MOF-74 electrode exhibited reduced capacitance, resulting in a decreased ECSA. This reduction can be attributed to structural changes after reconstruction under oxidative conditions, potentially leading to a decrease in accessible active sites and collapse of the microporous structure. The effects of EG concentration, pH, and temperature on the specific activity toward EGOR were also investigated. The catalytic current density displayed a linear increase with EG concentration, indicating a first-order rate dependence of EGOR on the concentration of EG (Figure S8a,b). The pH-dependent LSVs of NF|Co-MOF-74 demonstrated a cathodic shift of the catalytic onset potential and current enhancement with increasing pH, highlighting the beneficial role of the alkaline medium toward EGOR (Figure S9a). The onset potential required for the 10 mA cm^–2^ current density showed a linear dependence on pH with a slope of 111 mV pH dec^–1^ (Figure S9b), which suggests a 2H^+^/1e^–^ rate limiting step in EGOR. To determine the activation energy of EGOR on NF|Co-MOF-74, temperature-dependent LSVs were recorded. The Arrhenius plot for log *j* (current density) versus 1/T at 1.4 V demonstrated a linear correlation, and an activation energy of 25.2 kJ mol^–1^ was estimated from the slope of the linear fit of the data (Figure S10). This value is consistent with previous reports on EGOR using Pt catalysts and Co-based coordination polymers.^47,49^

### Controlled Potential Electrolysis for EGOR

To evaluate the oxidation products from EGOR, controlled potential electrolysis (CPE) was performed in 0.1 M EG at 1.40 V vs RHE using electrochemically conditioned NF|Co-MOF-74 (Figure 3). ^1^H NMR spectra of the electrolyte after CPE revealed a new peak at 8.46 ppm from formate, which was the primary product from EGOR. This was further confirmed by ion chromatography (IC). A steady decrease in current density was observed due to the consumption of EG, which was confirmed by ^1^H NMR (discussed later). Monitoring the concentration of formate by IC over the course of 4 h CPE revealed a continual decrease in FE from ∼100% after 30 min to ∼52% after 4 h. The chemical yield for EG-to-formate conversion reached a maximum of 78% after 2.5 h, which was followed by a gradual decrease in formate yield (Figure 3a, inset). Recycling the same electrode for multiple EGOR electrolysis experiments showed similar current density, formate yield, and Faradaic efficiency, which confirmed the robustness and stability of the restructured cobalt oxyhydroxide phase under operating conditions.

**Figure 3:**
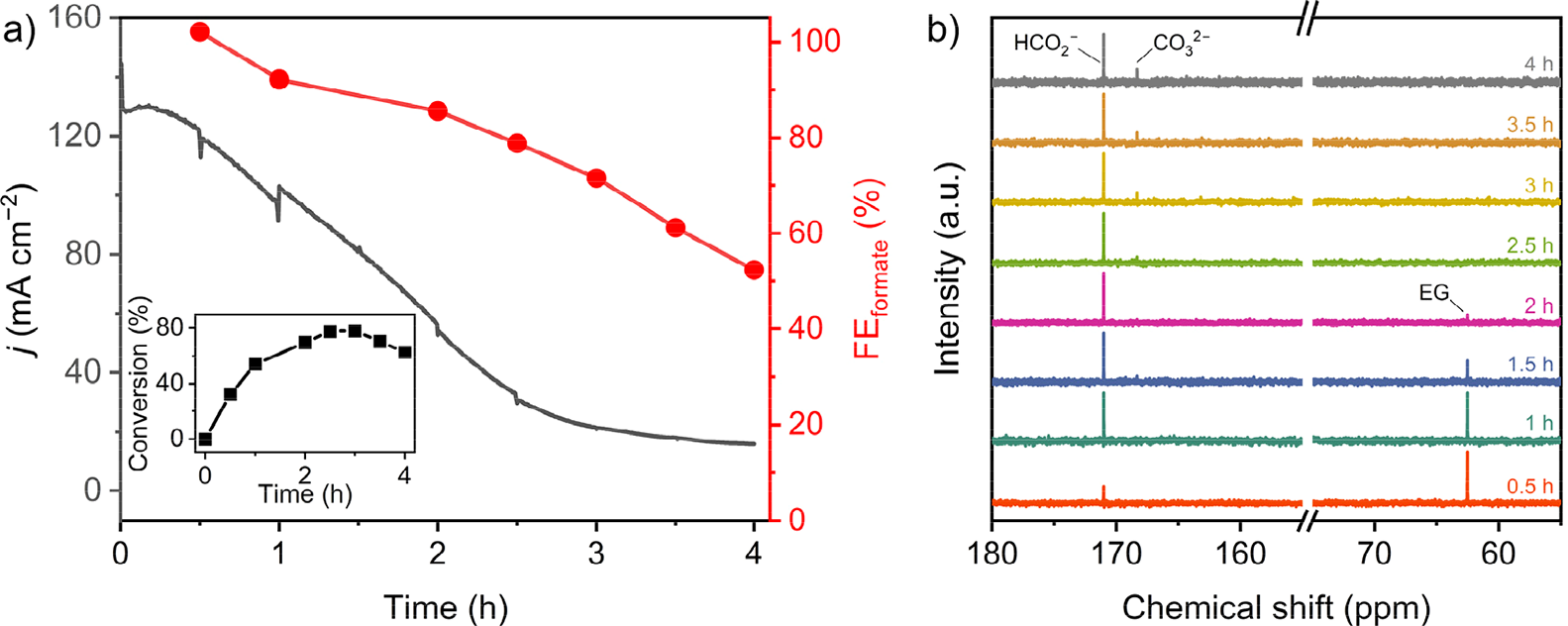
(a) Controlled potential electrolysis data for NF|Co-MOF-74 at 1.4 V vs RHE in 0.1 M EG in 1 M KOH. The black trace shows the current density against time and the red trace presents the Faradaic efficiency for formate generation. The inset figure shows EG-to-formate conversion over the course of CPE. (b) ^13^C NMR of the electrolyte showing formation of formate and carbonate.

Oxidation of 1 mol of EG to 2 mol of formate is a 6 e^–^ process, and when the CPE was performed with 1.5 mmol of EG, 858 C (8.9 mmol) charge was passed after 2.5 h. Quantification of the electrolyte using an internal standard in ^1^H NMR showed that >95% EG was consumed after 2.5 h CPE (Figure S11). Notably, the mass balance of the reaction showed a steady decrease from ∼94% after 0.5 h to ∼53% after 3.5 h (Figure S12). Further analysis of the electrolyte solution with ^13^C NMR showed the appearance of a new peak at 168 ppm after 2.5 h in addition to the formate peak at 171 ppm (Figure 3b). The new peak can be attributed to CO_3_^2–^, implying overoxidation of formate over longer CPE.^47^ The combined NMR and IC data indicate that NF|Co-MOF-74 can selectively oxidize EG to formate. To corroborate the overoxidation pathway, LSVs were performed in 1 M KOH in the presence of 0.1 M formate, which showed onset of the formate oxidation wave at ∼1.38 V (Figure S13). Although EGOR has an earlier onset potential on NF|Co-MOF-74 for selective conversion to formate, when EG was mostly depleted after 2.5 h and the formate concentration plateaued, the overoxidation pathway to carbonate became prominent. A trace amount of glycolic acid was detected by ^1^H NMR (δ = 3.9 ppm), which disappeared over the course of the electrolysis (Figure S14a). This suggests two plausible mechanisms for EGOR via either a glyoxal intermediate or a glycolic acid intermediate (Figure S14b).^18,20,30,48^ The proposed mechanism begins with the oxidation of EG to glycolaldehyde, which is subsequently oxidized to either glyoxal or glycolic acid. The glyoxal intermediate may undergo C–C bond cleavage to produce formate. Alternatively, glyoxal may also undergo Cannizzaro disproportionation under operational alkaline conditions, producing glycolic acid. Regardless of its origin, glycolic acid then undergoes oxidative C–C bond cleavage, leading to the formation of formate as the final product.

It should be noted that the tentative mechanism is proposed based on the detection of glycolic acid and previous reports on EGOR as no aldehyde intermediates were observed by ^1^H NMR. The detection of aldehydes by NMR is inherently challenging, particularly for short-lived or low-concentration species, which can undergo polymerization, precipitation, and disproportionation in alkaline conditions.^50^ Recent DFT studies on CoOOH-mediated EGOR have proposed a stepwise cascade oxidation pathway in which the oxidation proceeds via selective activation of one carbon site of ethylene glycol, delaying C–C bond cleavage until formic acid formation. In this mechanism, the overall reaction kinetics are governed by the generation and stabilization of OH* active species on the catalyst surface, highlighting that the intrinsic activity is dictated by the ability of the oxyhydroxide lattice to produce and adsorb these key intermediates.^25,51^

### Postcatalysis Characterization of the Electrode

We hypothesized that during electrolysis, Co-MOF-74 undergoes structural transformation due to the strongly oxidizing conditions in the alkaline electrolyte, which resulted in the change of the color of the catalyst. To gain an in-depth understanding, a range of characterizations was performed postcatalysis and under operating conditions for NF|Co-MOF-74 electrodes. SEM of the electrode after catalysis revealed roughening of the MOF particles and a loss of the clear hexagonal rod-shape morphology (Figure S15). Energy-dispersive X-ray spectroscopy (EDS) of the electrode postcatalysis showed an increase in the surface concentration of Co and O and a notable decrease in carbon content (Table S1). The increase in the level of O is consistent with the formation of metal hydroxide/oxyhydroxide species that likely play a key role in electrocatalysis. The increased Ni content in EDS can be attributed to exposure of the underlying NF substrate after electrolysis. Powder X-ray diffraction of the postcatalysis electrode showed a complete loss of the characteristic diffraction peaks for the Co-MOF-74 framework (Figure S16a). Additionally, the diffraction pattern of the detached catalyst from the NF substrate demonstrated the existence of a CoOOH phase (Figure S16b),^52^ which was further supported by TEM analysis (Figure S17).The SAED pattern and the FFT analysis from the HRTEM image displayed multiple diffraction rings and spots with lattice spacings of 0.42 (003), 0.24 (101), 0.23 (012), 0.21 (006), 0.14 (110), and 0.11 (205) nm, which are representative of crystallographic planes of the CoOOH phase (PDF no. 07-0169).^53^ This implies that *in situ* reconstruction of Co-MOF-74 takes place during electrolysis to form an amorphous active catalyst for EGOR.

To investigate the changes in the local electronic and atomic structures around Co centers, X-ray absorption spectroscopy (XAS) measurements were performed at the Co K-edge, including X-ray absorption near-edge structure (XANES) and extended X-ray absorption fine structure (EXAFS) analysis. The normalized Co K-edge XANES spectra are shown in Figure 4a. Relative to pristine Co-MOF-74, the edge peak near 7720 eV had been negatively shifted for soaked Co-MOF-74. Following catalysis, a clear shift of the absorption edge (*E*_0_) to higher energy was observed, demonstrating partial oxidation of the Co^2+^ centers in the material. Fitting the *E*_0_ values with the MOF samples with the two standard Co samples reveals Co-oxidation states of 2.4, 2.1, and 2.6 for the pristine, soaked, and postcatalysis materials (Figure S18). The Fourier transform of the Co K-edge EXAFS (*k* ^3^-weighted) spectrum of pristine Co-MOF-74 showed a dominant peak at 1.62 Å corresponding to the nearest-neighbor (Co–O bond) contribution and a weaker peak at 2.63 Å assigned to Co–Co single scattering paths (Figure 4b). After the electrode was soaked in KOH, a new peak emerged at 2.80 Å, while the first Co–O peak at 1.65 Å remained largely unaffected. The EXAFS data exhibit similarity to that of Co­(OH)_2_, in which the second peak is attributed to Co–Co scattering paths between the Co centers of di-μ-oxo bridged [CoO_6_] octahedra.^54^ The EXAFS data indicate that KOH-induced structural changes of Co-MOF-74 had a minimal impact on the first coordination shell (CoO_6_ octahedra) but resulted in the formation of a new Co–Co coordination shell. EXAFS of postcatalysis Co-MOF-74 demonstrated a shift of Co–O and Co–Co peaks from 1.67 to 1.53 Å and 2.77 to 2.51 Å, respectively, indicating shorter scattering paths. As shown in Figure 4b, the resulting EXAFS features are consistent with the formation of CoOOH after catalysis. The lower intensity of EXAFS peaks suggests that the postcatalysis material has a disordered local structure with a significant population of coordinatively unsaturated Co centers. It should be noted that the peak at <1 Å for postcatalysis sample (blue trace, Figure 4b) is an artifact from the Fourier transform of the experimental data, as the distance is too short for any interatomic scattering pathway.

**Figure 4:**
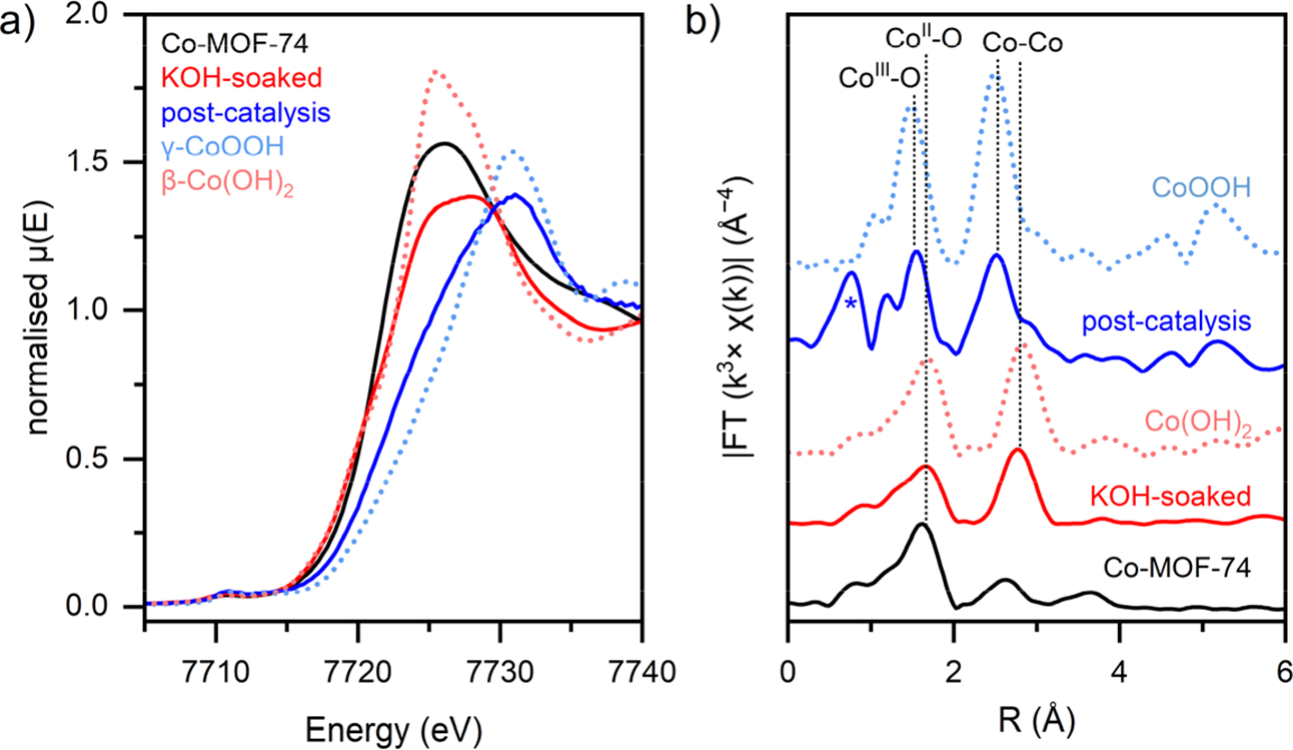
(a) Co K-edge XANES spectra and (b) Fourier-transformed *k* ^3^-weighted EXAFS signals for pristine Co-MOF-74, KOH-treated Co-MOF-74, and postcatalysis material compared with two cobalt reference compounds, β-Co­(OH)_2_ and γ-CoOOH. The artifact peak in the postcatalysis sample is marked by an asterisk.

This structural evolution was supported with XPS analysis of the KOH-soaked and postcatalysis electrodes (Figures 5a and S19 and S20). XPS spectra of the electrode after KOH treatment showed that the positions of Co 2p_3/2_ and 2p_1/2_ peaks remain largely unaffected (Figure 5a) with prominent satellite features at ∼786 and ∼802 eV, which is consistent with the high-spin Co^2+^ state in Co­(OH)_2_.^55^ In contrast, the postcatalysis electrode showed a clear shift of the Co 2p peaks to lower energy (778.1 and 793.1 eV) and significantly diminished intensity of the satellite peaks. These findings suggest that the Co centers are either completely or partially in a low-spin Co^3+^ state,^52,56^ attributable to *in situ* formation of cobalt oxyhydroxide species.^57^ *In situ* Raman spectroelectrochemistry was performed to further investigate the restructuring of Co-MOF-74 during electrolysis (Figure 5b). Raman spectra of the electrode in 1 M KOH with 0.1 M EG from open circuit voltage (∼+0.45 V) to +1.25 V showed gradual disappearance of the characteristic peaks for Co-MOF-74 and a new weak band arising at ∼490 cm^–1^. At potentials before the onset of catalysis (0.95–1.15 V), the two most intense vibrations at 1275 and 1415 cm^–1^ were still detectable, albeit at much diminished intensity, which completely disappeared at 1.25 V. This is consistent with restructuring of Co-MOF-74, leading to the loss of 2,4-dihydroxyterephthalate ligands and evolution of the cobalt (oxy)­hydroxide layer as the active catalyst. The vibration at ∼490 cm^–1^ can be tentatively assigned to Co–O vibrations in the newly evolved active catalyst layer.^52^

**Figure 5:**
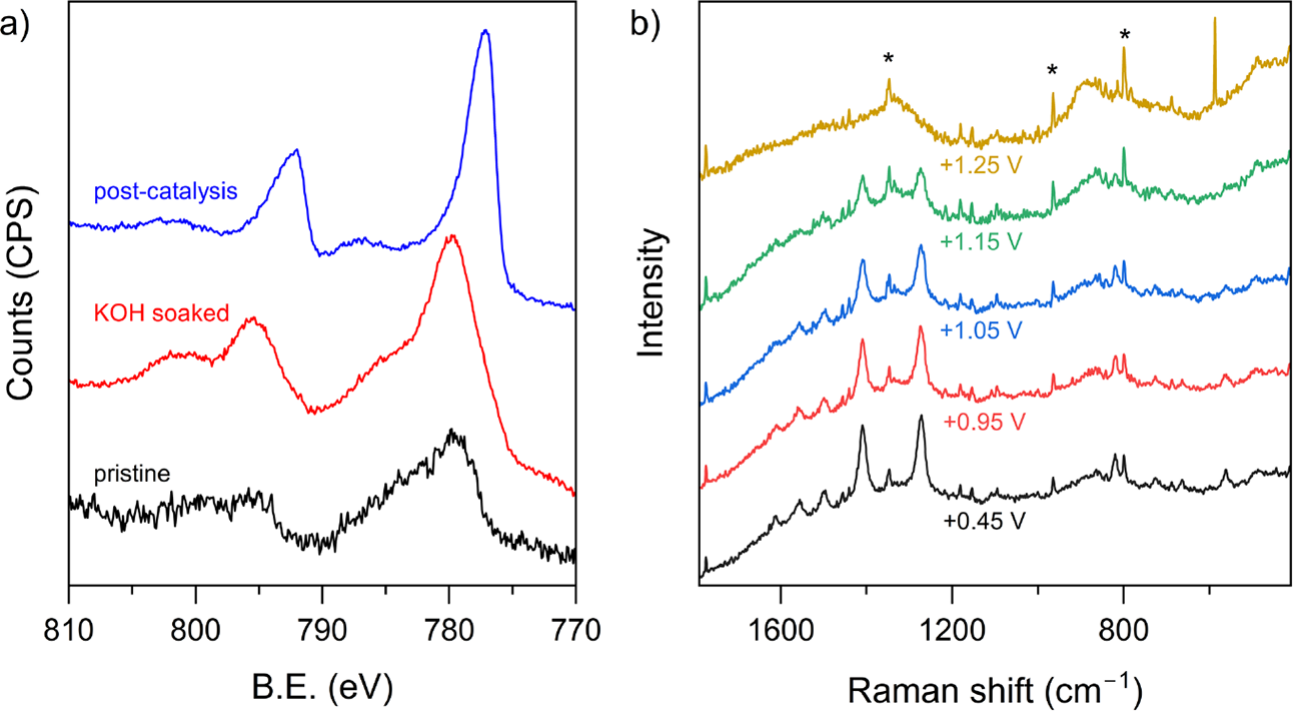
(a) Co 2p region of the XPS of pristine, electrolyte-soaked, and postcatalysis Co-MOF-74 deposited on the carbon paper electrode. (b) In situ Raman spectra of NF|Co-MOF-74 during the EGOR process with 1 M KOH containing 0.1 M EG. The applied potential range was +0.95 V vs RHE to +1.25 V vs RHE and the electrode was held at each potential step for 2 min before recording the spectrum. The artifacts in the spectra are marked with asterisks.

### Electrooxidation of PET Hydrolysate

The excellent electrocatalytic activity for EGOR displayed by NF|Co-MOF-74 prompted us to explore the electroreforming of PET. For PET upcycling into added-value products, commercial PET was depolymerized by alkaline hydrolysis for 48 h followed by direct utilization of the hydrolysate for CPE. Quantitative ^1^H NMR analysis of the PET hydrolysate in the presence of DMSO as internal standard showed characteristic peaks for terephthalic acid (singlet at 7.88 ppm) and EG (singlet at 3.66 ppm) with respective concentrations of 48 mM and 24 mM (Figure S21a). Interestingly, another set of multiplets were observed at 3.62 and 1.59 ppm with equal integration to EG. Based on the chemical shift in ^1^H and ^13^C NMR (Figure S21), this set of peaks can be attributed to 1,4-butanediol, which gives a concentration of 24 mM. The origin of this compound in PET hydrolysate can be tentatively attributed to the presence of poly­(butylene terephthalate) (PBT) impurities in the PET feedstock.

LSV of the PET hydrolysate showed a similar onset and catalytic behavior (Figure S22) to that of EGOR. To corroborate the electrooxidation products from PET hydrolysate, we carried out CPE experiments with a NF|Co-MOF-74 electrode at applied potentials ranging from 1.29 to 1.43 V vs RHE (Figure S23), which correspond to overpotentials (η_EGOR_) in the range of 1.04–1.18 V (*E*_EG/formate_^0^ = 0.254 vs RHE). Quantitative analysis of the electrolyte using ^1^H NMR and IC confirmed formate production, while ^13^C NMR was used to probe overoxidation to carbonate. Typically, the formate concentration in the electrolyte plateaued at a maximum value, followed by a gradual decrease in concentration, likely due to further oxidation of formate. The time required for the formate yield (% conversion from EG) to reach a plateau was strongly dependent on the applied potential (Figure S24). In the range of 1.29–1.39 V, a near unity Faradaic efficiency for formate (FE_formate_) was observed at the beginning, which underwent a steady decrease over the course of electrolysis. At 1.29 V, the amount of formate produced peaked at ∼300 μmol (∼83% EG conversion) after 5 h of electrolysis, corresponding to a FE_formate_ of 66%. Upon increasing the applied potential to 1.33 and 1.39 V, formate yield peaked after 2 h with a slightly improved FE_formate_ of 75% and 72% and EG conversion of 85% and 91%, respectively. However, at a further oxidizing potential of 1.43 V, it took only 1.5 h to produce a comparable amount of formate (∼305 μmol, 85% EG conversion), but a significantly lower FE_formate_ of 39%, suggesting a dominant overoxidation pathway and/or competitive side reaction at this potential. The electrolysis at 1.39 V was monitored by ^1^H and ^13^C NMR of the electrolyte to follow the formation of formate and carbonate. As shown in Figure S25, EG in the electrolyte was almost fully consumed after 2 h, and formate was observed as the only oxidation product. This was further corroborated by ^13^C NMR which showed the disappearance of the EG peak at 62.5 ppm and the appearance of the formate peak at 171 ppm (Figure S26). Interestingly, the other compound in the hydrolysate (^1^H NMR peaks at 3.62 and 1.59 ppm, and ^13^C NMR at 61.5 and 27.8 ppm) remained unchanged during the first 2 h of electrolysis. Only after EG was depleted, the peaks corresponding to this compound started to disappear with concomitant appearance of a new set of ^1^H peaks at 3.60 (triplet), 2.41 (singlet), 2.23 (triplet), and 1.80 (multiplet), and ^13^C peaks at 38.5, 38.3, and 34.8 ppm, suggesting formation of oxidized products (Figures S26 and S27). The singlet at 2.41 ppm suggests succinic acid formation, whereas the other ^1^H peaks are likely associated with 4-hydroxybutanoic oxidation intermediates.^58^ Conversely, ^1^H NMR analysis of the anolyte after electrolysis at 1.29 V showed no detectable oxidation products derived from this compound, even after complete EG consumption, indicating that its oxidation may be suppressed under milder conditions (Figure S28). During the later stage of the electrolysis (2–6 h), the formate signal in the NMR spectra grew weaker, consistent with its overoxidation to carbonate. The low FE_formate_ values obtained at the later stages of electrolysis could be attributed to these side reactions (Figure S23). The overoxidation of formate to carbonate was confirmed by ^13^C NMR of the electrolyte after 15 h of electrolysis at 1.43 V, showing the appearance of a new peak at 168.3 ppm (Figure S29a). The carbonate formation became a dominant pathway with depletion of EG. However, it should be noted that trace aerobic CO_2_ in the headspace can also contribute toward buildup of carbonate in the electrolyte, making its quantification difficult. Since the electrolyte was only purged with N_2_ before electrolysis, we could not exclude ingress of air into the H-cell over a longer duration. The ^1^H NMR spectrum of the electrolyte after electrolysis displayed a relatively small peak for glycolic acid at ∼3.9 ppm, in line with the data obtained for EGOR (Figure S29b).

### Coupled Electrolysis with CO_2_R

Having demonstrated the electrocatalytic activity of NF|Co-MOF-74 toward EGOR and oxidation of PET hydrolysate, we turned toward applying this electrode within a coupled electrolyzer. Reducing CO_2_ to syngas at the cathode presents a promising half-reaction for utilizing the electrons derived from oxidation of PET hydrolysate and producing value-added chemicals at both cathodic and anodic compartments simultaneously. A polymeric cobalt phthalocyanine catalyst (CoPPc) was selected for the CO_2_ reduction (CO_2_R) reaction at the cathode.^8,59^ While the exact mechanism for CO_2_ reduction by CoPc remains open to discussion, it is commonly accepted that the cycle is initiated by an irreversible reduction of Co^II^ to Co^I^, which is the resting state for cobalt. The reduced Co^I^ species then binds to CO_2_, forming a metal–CO_2_ adduct. Subsequent protonation of this adduct generates the CoPc-COOH* intermediate, which undergoes a proton-coupled electron transfer (PCET) to form CoPc-CO*. Finally, CO* is released from the metal center, regenerating the catalyst for the next cycle.^59–61^ To investigate coelectrolysis of CO_2_ and PET hydrolysate in a coupled system, we used a two-compartment H-cell separated by an anion-exchange membrane (AEM). A preconditioned NF|Co-MOF-74 electrode and CoPPc deposited on a carbon paper (CP| CoPPc) were used as the anode and cathode, respectively.

A three-electrode setup was initially adopted to test coupled electrolysis with EGOR and CO_2_R because it allows precise control of the applied potential at the working electrode against a known reference. A CO_2_-saturated 0.5 M NaHCO_3_ solution and a 1 M KOH solution containing 0.1 M EG were used as the catholyte and anolyte, respectively. CPE experiments were conducted at an applied potential of −0.58 V vs RHE (pH 7.3) over 3 h using CP|CoPPc as the working electrode (WE) and NF|Co-MOF-74 as the counter electrode (CE) (Figure S30). The potential at the counter electrode (*E*_CE_) gradually increased from ∼1.31 V to ∼1.46 V (vs RHE) over the course of 3 h electrolysis, indicating that the potential at the anode is sufficiently positive to drive EGOR (Figure S30d). This gives an overall cell voltage requirement in the range of 1.92–2.07 V for coupling the two half-reactions in an electrolyzer, as determined by the expression $\left|E_{cathode}-E_{anode}\right|\approx \left|{\mathrm{E̅}}_{CE}-E_{app}\right|$ . After 3 h of electrolysis, the CP|CoPPc cathode produced syngas (136 μmol of CO and 36 μmol of H_2_) with a cobalt-based total turnover number (TON) of 521 (TON_CO_ = 413 and TON_H2_ = 108) and an overall CO selectivity of 80%. The total Faradaic efficiency for syngas production (FE_CO+H2_) showed a steady decline from 86% to 67% over 3 h of electrolysis, while the CO:H_2_ ratio varied between 4.9 and 3.1, without any clear trend (Figure S30b,c). This could be attributed to the depletion of aqueous CO_2_ in the electrolyte as the postcatalysis XPS of the electrode showed that the molecular integrity of CoPPc was retained (Figure S31), in line with previous reports on CoPc-based cathodes.^62^ However, the inhibitory effect from bicarbonate poisoning of CoPc active sites cannot be completely excluded at *E*_app_ = −0.58 V vs RHE (−1.01 V vs SHE).^63^ At the anode, EGOR produced 145 μmol of formate after 3 h of electrolysis with a FE_formate_ of ∼85%, indicating that the overoxidation pathway is less severe in the coupled system.

The coupled system was further investigated in a more practical two-electrode configuration with CP|CoPPc as the working electrode and NF|Co-MOF-74 as the counter electrode. The applied cell voltage (*E*_cell_ = |*E*_CE_ – *E*_WE_ *|*) was varied in the range of 1.5 to 1.8 V, which was slightly lower than the potential difference observed during three-electrode measurements (∼1.9 to 2.0 V). The purpose was to minimize the overoxidation of formate by applying milder oxidative potentials. Chronoamperometric curves (*j* vs *t* plot) showed stable cathodic currents over 2 h electrolysis at all four applied *E*_cell_ values (Figure 6a). The syngas ratio (CO:H_2_) at the cathode was highly sensitive to the applied potential in Figure 6a (inset), which is consistent with the previous reports on CoPc-based electrocatalysts describing the dependence of CO selectivity on applied potential.^59,64^ The highest CO:H_2_ was observed at 1.8 V, while a low CO:H_2_ ratio (<1) was observed at 1.5 V. However, the combined FE for syngas formation at the cathode was higher at cell voltages of 1.6 and 1.7 V achieving 78% and 88%, respectively, compared to only 67% obtained at 1.8 V (Figure 6b). Interestingly, the FE_formate_ remained in the range 80–90% over the course of 2 h of electrolysis at all four applied voltages (Figure 6c). This can be attributed to relatively low conversion of EG to formate in the two-electrode configuration. The formate yield after 2 h of electrolysis was only 2.4% at 1.5 V and 4.5% at 1.8 V (Figure 6c inset). While the reaction rate was clearly slower due to the lower driving force from cell voltage, the electrooxidation reaction showed high selectivity, which demonstrates that the NF|Co-MOF-74 electrode performed better in a simpler 2-electrode electrolyzer configuration.

**Figure 6:**
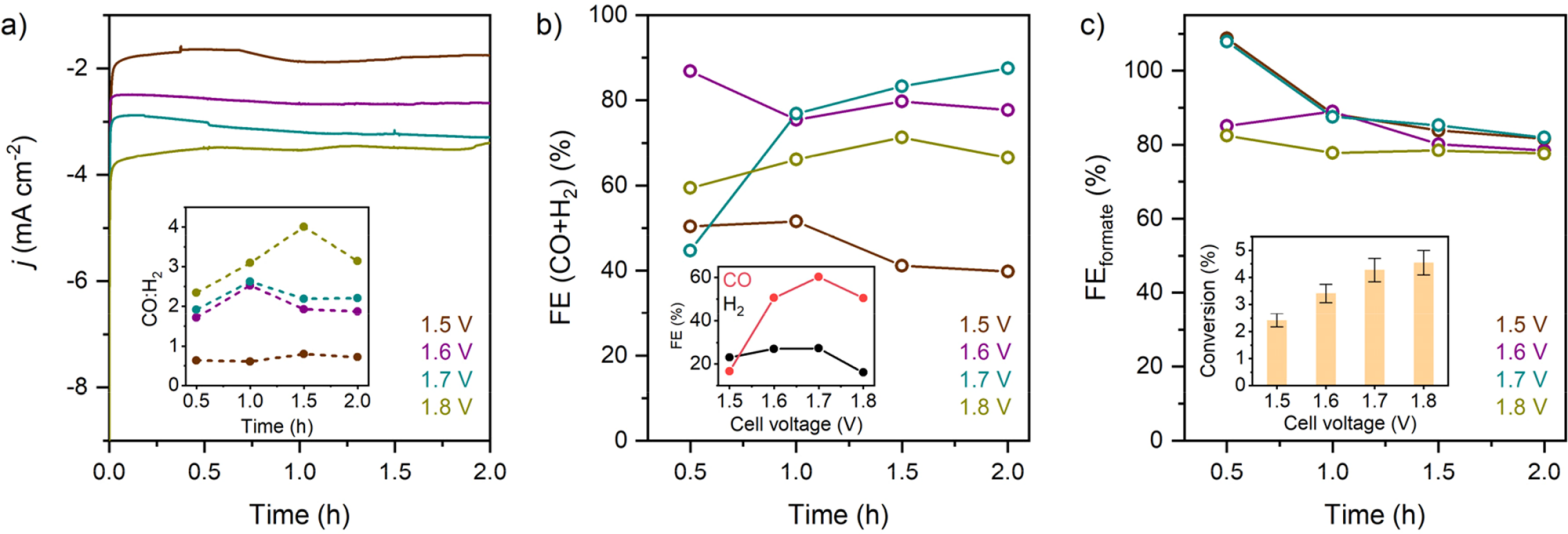
(a) Chronoamperometric curves of the CPE for the two-electrode configuration of the coupled electrolyzer at 1.5, 1.6, 1.7, and 1.8 V. CP|CoPPc was used at the working electrode and NF|Co-MOF-74 as the counter electrode. The inset shows the CO:H_2_ ratio over time at the different applied potentials. (b) Combined FE at the cathode over time at applied voltages of 1.5, 1.6, 1.7, and 1.8 V. The inset displays FE for the CO and H_2_ generated after 2 h of electrolysis. (c) FE_formate_ at the anode over time at applied voltages of 1.5, 1.6, 1.7, and 1.8 V. The inset figure shows EG-to-formate conversion after 2 h electrolysis at different cell voltages. Experimental conditions: two-compartment H-type cell separated by an AEM; anode: EG substrate (0.1 M) in KOH (1 M) solution; cathode: CO_2_-saturated NaHCO_3_ (0.5 M) solution. EGOR products and gas phase products were quantified by IC and GC, respectively.

This performance encouraged us to test the coupled electrolyzer in the two-electrode configuration using PET hydrolysate as the anolyte. Since the CO:H_2_ ratio was low at 1.5 V, the coupled electrolyzer with PET hydrolysate was only tested at 1.6, 1.7, and 1.8 V. Similar CO:H_2_ ratios were observed at the cathode after 2 h electrolysis with FE_CO+H2_ in the range of 60–78% (Figures S32 and S33). The chemical yield of formate (% conversion) in the anolyte increased from 11.7% to 15.8% and 17.8% for 1.6, 1.7, and 1.8 V, respectively. Across these potentials, FE_formate_ consistently exceeded 70%, exhibiting a maximum of 78% at 1.6 V (Figure S33). A trace amount of glycolic acid (GA) was detected in the anolyte by ^1^H NMR, suggesting a slight underestimation of the total anodic FE (Figure S34). The overall FE of the coupled electrolyzer peaked at 1.6 V with a combined FE of 156% (syngas and formate at the cathode and anode, respectively). To assess the performance of the coupled electrolyzer and benchmark it against reported systems, we estimated the energy efficiency (ε) based on the Gibbs free energy change for the overall reaction (Δ*G*_*rxn*_^0^) using the following formula (see Supporting Information for further details):^65,66^

$$\varepsilon =\frac{{\Delta G}_{rxn}^{0}}{nFE_{cell}}\times {FE}_{\left(CO+H_{2}\right)}\times {FE}_{HCOOH}\times 100\%$$

where *n* is the total number of electrons transferred per reaction cycle, *F* is the Faraday constant, *E*_cell_ is the operating cell voltage, ${FE}_{\left(CO+H_{2}\right)}$ is the total Faradaic efficiency for syngas production at the cathode, and *FE*_HCOOH_ is the Faradaic efficiency for formate generation at the anode. The highest cell energy efficiency (12.1%) was observed at *E*_cell_ = 1.6 V, which slightly outperforms the recently reported EGOR|CO_2_R electrolyzers (Table S3). At higher operating cell voltages, the energy efficiency decreases to ∼8% due to consumption of more electrical energy. However, it should be noted that the lower energy requirement for EGOR is included in the energy balance (Δ*G*_rxn_^0^) of the equation for ε, and as a result, the energy efficiency of the EGOR|CO_2_R electrolyzer might appear lower than conventional OER|CO_2_R due to larger overpotential applied for EGOR. This highlights the inherent trade-off in EGOR|CO_2_R electrolyzer design where EGOR provides a substantial saving in electrical energy consumption while delivering reduced thermodynamic efficiency.

### Coupled Electrolysis under Continuous Flow

To further highlight the advantages of coupled electrolysis, the system was tested in a gas-fed flow cell for CO_2_R which can overcome mass transport limitations of H-cells and reach higher current densities.^67^ In this arrangement, the flowing electrolyte solution (1 M KOH) was passed over the surface of a CP|CoPPc gas-diffusion electrode, while CO_2_ was supplied through the back of the electrode via a serpentine channel (Figure 7a). At the anode, the preactivated NF|Co-MOF-74 anode was connected to a Ni current collector and the compartment separated from the cathode chamber with an anion-exchange membrane (AEM, Sustainion-X37) to prevent reactant and product crossover (Figure 7a). In this setup, the addition of EG (0.1 M) decreased the half-cell voltage for the anode by ∼0.3 V for current densities between 10 and 100 mA cm^–2^, demonstrating that the anode retained high activity for EGOR under flow conditions (Figure 7b). This beneficial reduction in voltage resulted in corresponding full-cell voltages (*E*_cell_) ∼0.3 V lower than that of the OER-coupled device (Figure 7c). The FE_HCOO–_ between 25 and 75 mA cm^–2^ over short durations was high, but dropped slightly at 100 mA cm^–2^, which can be attributed to a greater susceptibility for overoxidation at higher potentials (Figure 7d). As expected from H-cell results, the FE_CO_ decreased with increasing current density; however, at 75 mA cm^–2^, a good FE_CO_ value of ∼70% was achieved (Figure 7d). Electrolysis was conducted at 75 mA cm^–2^ over 2 h, with results showing that a decay in selectivity for CO was observed over time but that the FE_HCOO–_ at the anode was consistently above 75% (Figure 7e). The loss of CO selectivity likely stems from flooding, which enhances the susceptibility for H_2_ evolution. The energy efficiency (ε) of EGOR|CO_2_R electrolysis in the flow-cell was determined to be 10.2% after 1 h of electrolysis at 75 mA cm^–2^. The higher operating cell voltage (∼2.3 V) in the flow cell was compensated by improved Faradaic efficiency for both cathodic and anodic reactions, compared to the H-cell.

**Figure 7:**
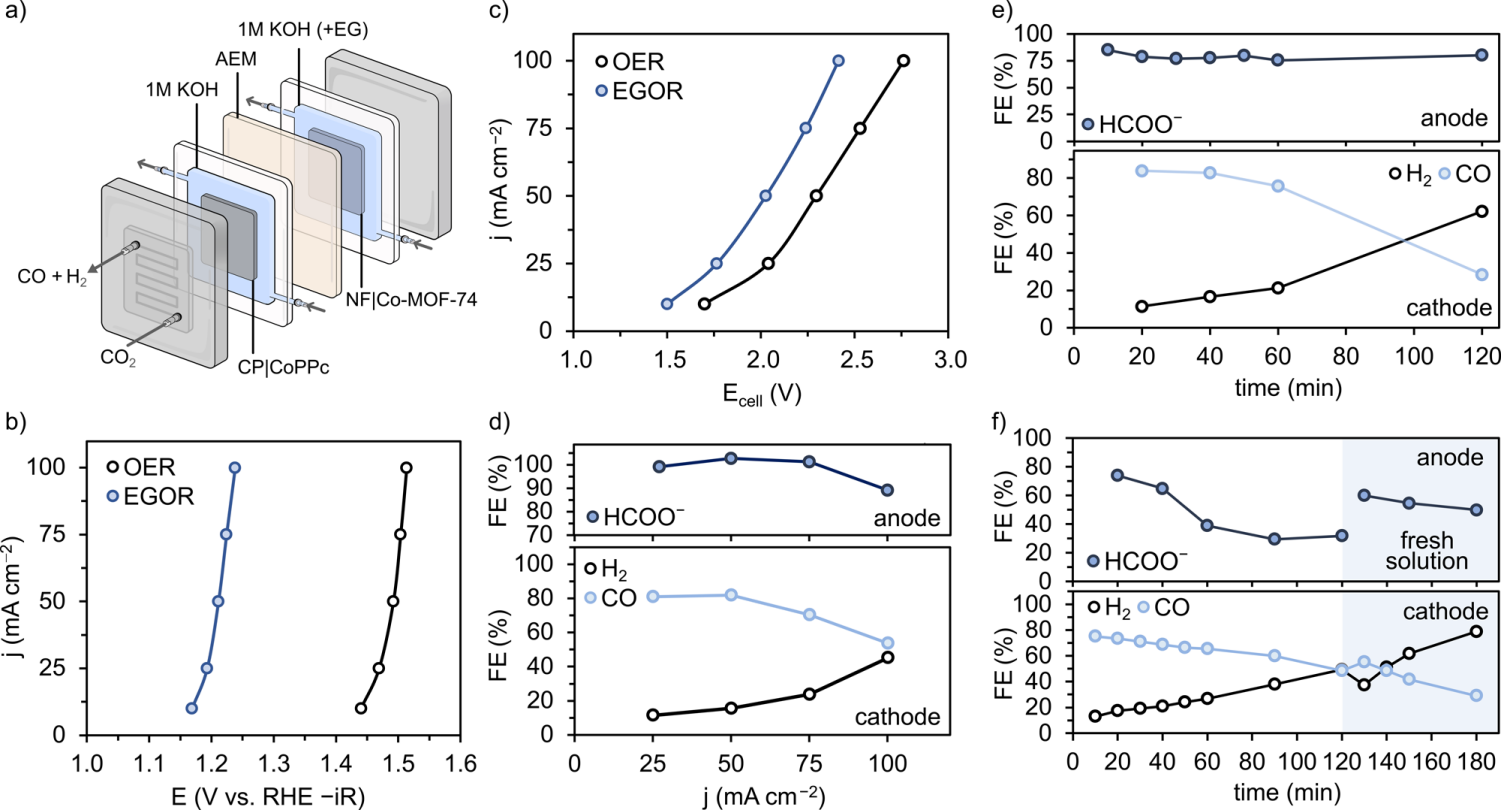
(a) Schematic of the gas-fed flow cell for coupled electrolysis where AEM is anion exchange membrane. (b) Half-cell (*iR*-corrected) and (c) full-cell current–voltage response for OER and EGOR in the flow cell obtained from chronopotentiometric steps. (d) FEs for anode and cathode products at different current densities (products taken at 5 min for the anode and 15 min for the cathode). (e) Time-dependent FEs for anode and cathode products for coupled CO_2_R and EGOR using 0.1 M EG at 75 mA cm^–2^. (f) Time-dependent FEs for anode and cathode products for coupled CO_2_R and oxidation of PET hydrolysate (in 2 M KOH) at 75 mA cm^–2^. Experimental conditions: anode: EG substrate (0.1 M) in KOH (1 M) solution or PET hydrolysate (∼60 mM EG) in KOH (2 M); cathode: 1 M KOH for EGOR or 2 M KOH for PET hydrolysate coupling. A CO_2_ flow rate of 20 mL min^–1^ and an electrolyte solution flow rate of 25 mL min^–1^ were maintained for all experiments. EGOR products and gas phase products were quantified by ^1^H NMR and GC, respectively.

The capabilities of the continuous flow cell were further outlined by introducing PET hydrolysate (in 2 M KOH) into the anode compartment, for which an *E*_cell_ of ∼2.3 V at 75 mA cm^–2^ was observed with simultaneous formate generation. The FE_CO_ at the cathode and FE_HCOO–_ at the anode gradually decreased over 2 h of electrolysis (Figure 7f). The drop in formate selectivity is more severe for the PET oxidation than EG, as the starting concentration is lower (∼60 mM) and there is potential for oxidation of any species derived from PBT impurities, as observed in the H-cell. However, repletion of the anolyte with fresh PET hydrolysate after 2 h increased selectivity back to >60%, highlighting that the activated anode retains high performance despite rapid depletion of EG. Further improvements to such systems will likely stem from improved cathodes alongside cell and process engineering to improve conversion for alternative oxidation reactions.^68^ Overall, this demonstration serves to illustrate that high reaction rates for CO_2_ electrolysis can be achieved with lower *E*_cell_ in a continuous flow reactor by replacing the OER with PET recycling.

## Conclusions

In summary, this work presents a comprehensive investigation of the irreversible structural transformation of Co-MOF-74 into a robust cobalt oxyhydroxide phase that displays high activity toward EGOR and electrochemical recycling of PET hydrolysate. In parallel, we demonstrate the utilization of PET electrooxidation reaction toward driving CO_2_ reduction with improved energy efficiency. Under electrolysis conditions, Co-MOF-74 undergoes structural reconstruction to form cobalt (oxy)­hydroxides, as confirmed by a combination of in situ and ex situ characterization techniques. In the presence of EG, the resulting CoOOH phase exhibits high activity toward EGOR, lowering the catalytic onset potential by 0.23 V compared with the OER at 100 mA cm^–2^. Coupling EGOR with CO_2_R in an integrated non-noble metal electrolyzer enabled concurrent valorization of PET and CO_2_R. In a three-electrode setup, syngas and formate were produced at the cathode and anode, respectively, with Faradaic efficiencies of 67% and 85% after 3 h. In a two-electrode configuration using PET hydrolysate as the anolyte, the integrated system achieved a combined Faradaic efficiency of 156% at 1.6 V after 2 h with an overall energy efficiency of 12.1%. A decrease in energy efficiency was observed at higher cell voltages due to increased consumption of electrical energy. Flow-cell experiments further demonstrated a 10.2% energy efficiency and a consistent ∼ 0.3 V reduction in full-cell voltage compared to systems employing OER, highlighting the lower energy demands of the CO_2_R-EGOR coupled electrolyzer. The paired electrolyzer demonstrates comparable performance metrics and energy efficiency to the benchmark systems, while offering a unique approach toward design of the anode catalyst.^23,24,66^ It presents a rare example of elucidating the structural reconstruction of the anode catalyst under operating conditions that plays a key role in controlling the EGOR activity. The MOF-derived catalyst largely outperforms a standard cobalt (oxy)­hydroxide catalyst prepared by an electrodeposition method, which highlights the unique benefit of using microporous Co-MOF-74 as a sacrificial scaffold for in situ generation of the active catalyst layer. We speculate that the high surface area, microporous structure, and morphology of the particles could be the contributing factors, but further investigations are required to understand the origin of superior activity. Notably, it paves the way for the rational design and postsynthetic reconstruction of MOFs to develop efficient catalysts for electrochemical upcycling of diverse waste streams. However, further in-depth structure–activity analysis using different MOF templates and theoretical investigation to support the mechanism will be necessary to expand the scope of catalysts and new electrooxidation reactions. Finally, coupling EGOR as an alternative anodic reaction with CO_2_R in a non-noble metal CO_2_ electrolyzer offers significant potential for enhanced productivity and economic benefits through the simultaneous generation of valuable chemicals.

## Experimental Section

### Synthesis of Co-MOF-74

CoCl_2_·6H_2_O (0.38 mmol), 2,5-dihydroxyterephthalic acid (0.13 mmol), DMF (5 mL), and deionized water (0.25 mL) were mixed in a vial and sonicated until complete dissolution of the solids. A clean nickel foam electrode was placed inside the reaction mixture, and the vial was heated at 100 °C for 24 h. After the synthesis, NF|Co-MOF-74 electrodes were soaked in DMF and methanol for 12 h and dried in air. The solid Co-MOF-74 was collected by filtration and washed thoroughly with DMF and methanol and dried in air. To prepare NF|MOF-74 electrodes, a cleaned NF electrode was placed into the vial containing the MOF synthesis solution and then placed in the oven for 24 h at 100 °C. Following the synthesis, Co-MOF-74-coated NF electrodes were removed from the vials, washed with DMF and methanol, and dried under a stream of N_2_.

### Synthesis of NF|Co­(OH)_2_

NF|Co­(OH)_2_ was prepared by electrodeposition following a previously reported procedure.^25^ In brief, a cleaned NF electrode was immersed in a 0.2 M Co­(NO_3_)_2_ aqueous solution and subjected to a constant potential of −0.956 V versus Ag|AgCl for 300 s. The resulting NF|Co­(OH)_2_ was electrochemically activated by performing 50 cyclic voltametric scans from 0 to 0.8 V vs Ag|AgCl. The resulting electrode was used to record the linear sweep voltammograms for EGOR.

### Polymeric Cobalt Phthalocyanine (CoPPc) Hybrid Electrode Preparation

Polymeric cobalt phthalocyanine (CoPPc) on multiwalled carbon nanotubes (MWCNTs) was synthesized by scaling up a previously reported method.^59^ In a typical synthesis, 40 mg of MWCNTs were dispersed in 10 mL of pentanol by sonicating for 30 min. To this dispersion, 1,2,4,5-tetracyanobenzene (40 mg, 0.44 mmol), CoCl_2_·6H_2_O (26.4 mg, 0.11 mmol), and 1,8-diazabicyclo[5.4.0]­undec-7-ene (33.6 μL, 0.22 mmol) were added. The resulting dispersion was purged with N_2_ for 10 min. Subsequently, the mixture was heated in a microwave reactor (Monowave 400, Anton Paar) at 180 °C for 2.5 h. The final MWCNT-CoPPc was collected by filtration, washed with ethanol, chloroform, 5% HCl solution, ethanol, and water, and dried under a vacuum. Cobalt loading was determined by inductively coupled plasma-optical emission spectroscopy (ICP-OES) as 0.66 mmol g^–1^. The hybrid electrode was prepared by drop-casting 100 μL of catalyst ink onto a 1 × 1 cm^2^ carbon paper electrode (AvCarb GDS2120). The ink was prepared by dispersing 5 mg of MWCNT-CoPPc and 30 μL of 5% Nafion solution in 1 mL of isopropanol with sonication for 1 h. The final catalyst loading was approximately 0.5 mg (0.33 μmol Co) per electrode. The cathode for use in the gas-fed flow cell was prepared by drop-casting 500 μL of a suspension containing 3 mg of MWCNT-CoPPc dispersed in 1 mL of EtOH and 15 μL of 5% Nafion solution (sonicated for 1 h) onto a 2 cm^2^ area of carbon paper electrode (AVCarb GDS5130) and heated at 60 °C. For each electrode, the catalyst loading was approximately 0.8 mg cm^–2^.

### PET Hydrolysis

PET was depolymerized by dispersing 2 g of predried PET in 100 mL of 2 M KOH solution. The mixture was heated to 60 °C with continuous stirring for 48 h. Afterward, the resulting cloudy solution was filtered by using a PTFE-H syringe filter (0.45 μm). A 120 μL aliquot of the PET hydrolysate was analyzed by ^1^H NMR to quantify the TPA and EG monomers in the solution using DMSO as the internal standard.

### Electrochemical Measurements

Electrochemical tests were carried out in an alkaline electrolyte (1 M KOH) using a three-electrode system on a BioLogic VSP potentiostat. For the EGOR, the electrolyte contained 0.1 M EG. A Ag|AgCl|KCl (satd.) reference electrode (CHI111) was used for all experiments. Pt-mesh or NF was used as the counter electrode during EGOR. NF|Co-MOF-74 electrodes were conditioned in an airtight, one-compartment cell using a three-electrode configuration. Controlled potential electrolysis experiments for investigating EGOR were performed in a two-compartment cell separated by an anion exchange membrane (Fumasep FAA-3-50) under ambient conditions. The Ag|AgCl|KCl (satd.) reference electrode was regularly calibrated against a master reference electrode, and any drift in the potential (*E*′) of the Ag|AgCl reference was corrected using the equation below (for Ag/AgCl/KCl_3.4M_, a correction of 0.206 was used instead of 0.197):

$$E_{corrected}\left(V\;vs\;Ag\left|AgCl)=E(V\;vs\;Ag\right|AgCl\right)+E^{\prime }$$

The reversible hydrogen electrode (RHE) potentials were obtained with the following equation:

$$E\left(V\;vs\;RHE\right)=0.197+E_{corrected}\left(V\;vs\;Ag|AgCl\right)+0.059\times pH$$

All *iR*-correction was conducted by using *R*_s_ values obtained from electrochemical impedance spectroscopy. The geometric surface area of the electrode was used for reporting the normalized current densities.^69^

The surface coverage of redox species (Γ) was estimated from the integration of the cathodic wave in the CV scan:

$$\Gamma =\frac{Q}{zFA}$$

where *Q* is the charge obtained from the integration of the cathodic wave, *z* is the number of electrons involved in the redox process (assumed to be 1), *F* is the Faraday constant (96,485 C mol^–1^), and *A* is the geometric surface area of the electrode (assumed to be 1 cm^2^). The number of electroactive redox species was estimated as the average charge obtained from the integration of the last 20 CV scans recorded during electrode conditioning (*Q* = 688.8 mC cm^–2^).

The percentage of electroactive species was calculated based on the loading of Co species in NF|Co-MOF-74 (*n*_Co_: calculated using ICP of the digested MOF films) and the calculated surface coverage of redox-active species (Γ_Co_):

$$\%=\frac{\Gamma_{Co}}{n_{Co}}\times 100$$

Tafel slopes were calculated from an LSV performed at 1 mV s^–1^ utilizing the Tafel equation: η = *b* log­(*j*) + *a*, where η denotes the overpotential, *j* denotes the current density, *a* is a constant, and *b* is the Tafel slope. All electrochemical experiments were carried out at room temperature (20 °C) unless otherwise stated. The electrochemical active surface area (ECSA) was calculated by recording CVs at variable scan rates (50–10 mv s^–1^) in the non-Faradaic region from −0.234 to −0.3340 V (vs RHE). The double-layer capacitance (*C*_dl_) is obtained by plotting the capacitive currents at −0.284 V, with the *C*_dl_ equaling half the gradient.

The coupled electrolysis under continuous flow was carried out in a gas-fed flow cell (Sphere Energy Ltd., Figure 7a) using a CP|CoPPc cathode, an activated NF|Co-MOF-74 anode (as previously described), a leak-free Ag|AgCl|KCl_3.4M_ (Innovative Instruments, Inc.) reference electrode, and an anion exchange membrane (AEM, Sustainion-X37) to separate the anolyte and catholyte. All experiments in flow were conducted using a Biologic SP-150e potentiostat. In this configuration, CO_2_ is continuously supplied at the back of the CP|CoPPc gas diffusion electrode through a serpentine channel at 20 mL min^–1^ by using a Bronkhorst mass flow controller. The catholyte and anolyte flow rates were maintained at a rate of 25 mL min^–1^. Different current densities were applied (10, 25, 50, 75, and 100 mA) for the chronopotentiometric steps (1.5 min) with sample collection for product quantification every 5 min at the anode and 20 min at the cathode. For the anolyte (100 mL), an EG substrate (0.1 M) in KOH (1 M) solution or PET hydrolysate in KOH (2 M) was used, while the catholyte (100 mL) was 1 M KOH for EGOR or 2 M KOH for PET hydrolysate coupling.

For product quantification from EGOR, 50 μL of electrolyte was diluted to 5 mL with deionized water and analyzed by ion chromatography. A representative IC trace and the calibration curve are shown in Figure S35. For NMR analysis, 50 μL of electrolyte was diluted with 550 μL of D_2_O and 5 μL of DMSO was added to the mixture as internal standard. For the CO_2_ reduction products, the gaseous products (H_2_ and CO) were quantified using gas chromatography (SRI 8610C) by injecting 50 μL of headspace gas. The CO and H_2_ peaks were determined by calibration with standard CO and H_2_ gas. The SRI gas chromatograph (multiple gas analyzer #1) is equipped with a thermal conductivity detector (TCD) and a flame ionization detector (FID) with a built-in methanizer attachment. A silica gel column (6 ft) was used to block CO_2_ and H_2_O, and a molecular sieve 13X (6 ft) main column was used to separate H_2_ and CO. N_2_ was used as the carrier gas at a 25 psi pressure. For the coupled flow electrolysis, the anolyte was analyzed using NMR (Bruker Ascend 400) for the quantification of EGOR and PET hydrolysate oxidation products. To prepare the NMR samples, 400 μL electrolyte was diluted with 100 μL D_2_O followed by addition of 100 μL DMSO (10 mM) as an internal standard. Gas products were detected online using an Agilent 8860 GC with an Ar carrier gas. An argon bleed was used to purge the catholyte solution and mixed with direct outlet gases from the electrochemical cell to ensure all products were detected while two flow meters (MesaLabs Defender 530+ and Ellutia 7000) were used to detect the flow after the cell for accurate FE calculations as in prior work.^70^ H_2_ detection was conducted using a TCD and CO was detected using an FID with a methanizer (ARC Jetanizer, Speck and Burke). The gases were separated using a HayeSep Q (8 ft) and an additional MolSieve 5A (6 ft) column for CO. An external calibration was used for product quantification, which was performed using a standard gas mixture in CO_2_ under flow conditions.

The Faradaic efficiency (FE) for the gas products was calculated using the equation below:

$$FE\;\left(\%\right)=\frac{n_{product}\times n_{electrons}\times F}{\left(Q_{t=0}-Q_{t=x}\right)}$$

where *n*_product_ represents the amount of H_2_ or CO detected in mol, *n*_electrons_ is the number of electrons used to make H_2_ (2e^–^) or CO (2e^–^), *F* is the Faraday constant (C mol^–1^), *Q*_*t*=0_ is the charge at the time of the injection, and *Q*_*t*=*x*_ is the charge at time *x* seconds before the injection, representing the time taken to fill the sample loop and *x* being dependent on the combined flow rates of the Ar bleed and CO_2_ and the loop size.

For Raman spectroelectrochemistry, the spectra were collected over the 410–1790 cm^–1^ spectral range at 25 mW power with a 2 s exposure time and 10 accumulations. NF|Co-MOF-74 was used as the working electrode, Ag/AgCl as the reference electrode, and a Pt wire as the counter electrode. During chronoamperometry, the electrode was held at the applied potential for 2 min before recording the spectrum. The spectroelectrochemical cell was purchased from Dek Research and the electrochemical measurements were performed using a PalmSens4 potentiostat with Bluetooth connectivity.

UV–vis spectroelectrochemistry was performed using a carbon paper electrode coated with NF|Co-MOF-74 (AvCarb P50T), an Ag|AgCl|KCl (3M) reference electrode, and a Pt wire counter electrode in a 1 mm path length glass cuvette. Before applying the oxidative potentials, the electrode was equilibrated for 5 min at the open-circuit potential in the electrolyte (1 M KOH). A deuterium and halogen light source (AvaLight-DHC) was used, an AvaSpec-2048 was used to record the spectra, and the UV–vis spectra were recorded every 30 s. The electrodes were prepared by drop casting 0.05 mL of catalyst-ink on carbon paper (AvCarb P50T) over an area of 1 × 1 cm^2^. The catalyst-ink used was prepared by dispersing 5 mg of Co-MOF-74 in 0.5 mL of IPA and 40 μL of 5 wt % Nafion solution.

## Supplementary Material



## Notes

Materials and methods details, additional figures including structural and spectroscopic characterization (PXRD, TEM, SEM, in situ UV–vis SEC, XAS, and XPS), electrochemical measurements (LSV, CV, Tafel plot, and EIS analysis), additional electrolysis data, product quantification via ^1^H NMR, qualitative product identification by ^1^H and ^13^C NMR, proposed reaction mechanism, graphical overview of the EGOR–CO_2_R coupled electrolyzer process, and IC calibration, and supplementary tables (PDF)
