# Supplementary material for: Coelectrolysis of PET and CO2 Using an Electrochemically Restructured Co-MOF-74 Anode and a Polymeric Co-Phthalocyanine Cathode
Source: ACS Appl Mater Interfaces. 2026 Jan 20;18(4):6937–51. doi: 10.1021/acsami.5c22269 (PMC12884455; doi:10.1021/acsami.5c22269)
Supplement: Supplementary file 1 [file am5c22269_si_001.pdf]

Supporting Information

Co-electrolysis of PET and CO_2 using an electrochemically restructured Co-MOF-74-anode and a polymeric Co-phthalocyanine cathode

Raúl Rojas-Luna,[1,3] Lewis S. Cousins,[2] Rhiannon Germaney,[1] Dolores G. Gil-Gavilán,[3] Miguel Castillo-Rodríguez,[4] Dora-Alicia Garcia Osorio,[2] Thomas Doughty,[1] Dolores Esquivel,[3] Charles E. Creissen,[2]* Souvik Roy[1]*

[1] Department of Chemistry, School of Natural Sciences, University of Lincoln, Green Lane, Lincoln LN6 7DL, U.K.

[2] School of Chemical and Physical Sciences, Keele University, Staffordshire, ST5 5BG, UK.

[3] Departamento de Química Orgánica, Instituto Químico para la Energía y el Medioambiente (IQUEMA), Facultad de Ciencias, Universidad de Córdoba, Campus de Rabanales, Edificio Marie Curie, 14071 Córdoba, Spain

[4] Departamento de Física Aplicada, Radiología y Medicina Física, Universidad de Córdoba, Campus de Rabanales, 14071 Córdoba, Spain.

e-mail: c.e.creissen@keele.ac.uk and sroy@lincoln.ac.uk

Materials and Methods

Chemicals. Cobalt chloride hexahydrate, ethanol (EtOH, ≥ 99.8%), acetone (≥99.8%), 1,8-diazabicyclo[5.4.0]undec-7-ene (DBU) and hydrochloric acid (HCl, ~37%) were purchased from Fisher Scientific. Nafion™ 117 solution (~5% in a mixture of lower aliphatic alcohols and water) and multiwalled carbon nanotube (>90% carbon basis, D × L 110-170 nm × 5-9 μm) were purchased from Merck. Cobalt(II) dihydroxide (97%) was purchased from Thermo Fisher Scientific. N,N-Dimethylformamide (DMF, Peptide grade) was purchased from Rathburn Chemicals. 1,2,4,5-tetracyanobenzene and 2,5-dihydroxyterephthalic acid were purchased from Fluorochem (Doug Discovery). An Elga Purelab purification system was used for all deionized (DI) water (15 MΩ cm at 22 °C). Nickel foam (NF) was purchased from Nanographenex (>99.99%, 1.6 mm thickness, surface density 304 g m^{-2}, ≥95% porosity). Carbon paper was purchased from the Fuel Cell Store (AvCarb P50T and AvCarb GDS2120) and Dioxide Materials (AVCarb GDS5130). Cobalt oxyhydroxide was synthesised using a literature method.[1]

Characterisation. Raman measurements on powder samples and electrodes were performed using a Bruker Senterra benchtop Raman microscope using 785 nm laser excitation and a 20× objective lens. The spectrometer was calibrated using a polystyrene standard. Fourier-transform infrared spectroscopy (FTIR) was performed on a Bruker Alpha II using ATR accessory. Powder X-ray diffraction data was collected on a Bruker D8 Discover using a Cu Kα radiation source (λ=1.54178 Å). X-ray photoelectron spectroscopy (XPS) was performed on a Thermo Fisher Scientific K-alpha+ spectrometer. Samples were analysed using a micro-focused monochromatic Al X-ray source over an area of approximately 200 microns. Data were recorded at pass energies of 150 eV for survey scans and 40 eV for a high-resolution scan with 1 eV and 0.1 eV step sizes, respectively. Data analysis was performed in CasaXPS using a Shirley type background and Scofield cross sections, with an energy dependence of –0.6. ICP measurements were performed using a Thermo Scientific iCAP 7000 series ICP spectrometer along with the Qtegra software. A multielemental standard solution 6 (Sigma Aldrich) was diluted to perform calibration in the range of 1-50 ppm. Each sample was measured in triplicates. The morphology of the both the electrodes and bulk powder materials were characterised using scanning electron microscopy (SEM Tescan Essence) with energy dispersive X-ray spectroscopy (EDX). Transmission electron microscopy (TEM) and high-resolution TEM (HRTEM) images were collected on a FEI Talos F200i S/TEM microscope operating at 200 kV using a spot size of 3. For TEM analysis, Co-MOF-74 was peeled off the NF by sonicating the electrode in isopropanol. After evaporation of the solvent, the sample was deposited onto carbon-coated copper grids (Agar Scientific Ltd.). NMR spectra were recorded on a Bruker Avance III HD 500 MHz instrument. IC was performed using a Dionex ICS-1100 system equipped with a conductivity detector and a Dionex AS-DV carousel autosampler set up (delivery speed of 4 mL/min). The samples were analysed using Na_2CO_3 (4.5 mmol L^{-1}) and $NaHCO_3$ (1.4 mmol L^{-1}) aqueous solution as the anion eluent with a flow rate of 1.20 mL/min and a suppressor current of 31 mA. A methanesulphonic acid solution (20 mmol L^{-1}) was used as the cation eluent and had a flow rate set to 1.00 mL min^{-1} and a suppressor current of 59 mA. The temperature was maintained at 35 °C with a run time of 16 min per sample.

X-ray absorption spectroscopy: XAS data was collected at beamline B18 at the Diamond Light Source (beamtime number SP-34632-1). Pellets (13mm) for pristine and KOH-soaked Co-MOF-74 were prepared by homogeneously mixing the MOF powder with an inert cellulose matrix and mounted onto a sample holder using Kapton tape. For analysis of a post-catalysis sample, 0.25 mL catalyst ink containing 16 mg Co-MOF-74 dispersed in 2 mL IPA and 0.04 mL 5 wt% Nafion solution using 30 min sonication, was drop casted onto 1 × 1 cm^2 carbon paper electrode (AvCarb GDS2120). The electrode was subjected to 6 h controlled potential electrolysis at 1.5 V. For XAS measurements the electrodes were cut into four 0.25 cm^2 squares and stacked using Kapton tape. Measurements of the electrodes were performed in fluorescence mode (6 scans), while the pellets were measured in transmission mode (3 scans). $Co(OH)_2$ (97%, Thermo Scientific Chemicals) and CoOOH were used as the cobalt standards. XAS data processing and EXAFS analysis were performed using Athena and Artemis software, respectively.

Calculation of the apparent activation energy

The apparent activation energy can be determined from the Arrhenius equation:

$$k(T) = Ae^{-\frac{E_a}{RT}} \tag{1}$$

$$\ln(k) = \ln(A) - \frac{E_a}{R} \cdot \frac{1}{T} \tag{2}$$

where k(T) is the rate constant, A is the pre-exponential factor, T is the temperature and R is the gas constant.

The apparent activation energy (E_a) for EG oxidation was estimated from temperature dependent LSVs recorded at different temperature, where Arrhenius plots are obtained by plotting ln j against the reciprocal of T.

$$E_a = -R\left[\frac{d\ln(j)}{d(1/T)}\right] \tag{3}$$

Coupled electrolysis and cell energy efficiency calculation

The overall process for the coupled electrolyser is composed of EGOR (anode) and the CO_2R (cathode) cathode whose both half-reactions can be expressed as follows. For the overall reaction, we considered syngas formation at the cathode with a $CO:H_2$ ratio of 2:1.

Anode: $C_2H_6O_2 + 6OH^- \rightarrow 2HCOOH + 4H_2O + 6e^-$ (4)

Cathode: $2CO_2 + 2H_2O + 4e^- \rightarrow 2CO + 4OH^-$ (5)

$2H_2O + 2e^- \rightarrow H_2 + 2OH^-$ (6)

Overall reaction: $C_2H_6O_2 + 2CO_2 \rightarrow 2HCOOH + 2CO + H_2$ (7)

The energy efficiency (ε) is defined as the ratio between the theoretical Gibbs free energy change of the overall reaction and the actual electrical energy input during electrolysis:

$$\varepsilon = \frac{\Delta G^0_{rxn}}{nFE_{cell}} \times FE_{(CO+H_2)} \times FE_{HCOOH} \times 100\% \tag{8}$$

The free energy change for the overall reaction (ΔG^0_{rxn}) is given by the following equation (Gibbs free energy values were obtained from Energy Environ. Sci., 2020,13, 472-494)[2]:

$$\Delta G^0_{rxn} = \Delta G^0_{f\,products} - \Delta G^0_{f\,reactants} = 2 \times \Delta G^0_{HCOOH} + 2 \times \Delta G^0_{CO} + \Delta G^0_{H_2} - \Delta G^0_{EG} - 2 \times \Delta G^0_{CO_2}$$

$$\Delta G^0_{HCOOH} = -361.4\ kJ\ mol^{-1}$$

$$\Delta G^0_{CO} = -137.2\ kJ\ mol^{-1}$$

$$\Delta G^0_{H_2} = 0\ kJ\ mol^{-1}$$

$$\Delta G^0_{EG} = -394.3\ kJ\ mol^{-1}$$

$$\Delta G^0_{CO_2} = -394.4\ kJ\ mol^{-1}$$

$$\Delta G^0_{rxn} = 185.9\ kJ\ mol^{-1}$$

$$\Delta E^0_{rxn} = 0.32\ V$$

The energy efficiency of the H-cell based paired-electrolyser at various applied voltages ($E_{cell} = 1.6, 1{,}7, 1.8\ V$) are shown below.

$$\varepsilon_{1.6\,V} = \frac{0.32}{1.6} \times 0.78 \times 0.78 \times 100\% = 12.1\%$$

$$\varepsilon_{1.7\,V} = \frac{0.32}{1.7} \times 0.58 \times 0.72 \times 100\% = 7.9\%$$

$$\varepsilon_{1.8\,V} = \frac{0.32}{1.8} \times 0.6 \times 0.77 \times 100\% = 8.2\%$$

However, it should be noted that the CO:H_2 ratio in the syngas produced at the cathode varies with applied voltage.

For the flow-cell configuration, the cell voltage (E_{cell}) was 2.3 V for 1 hour electrolysis at 75 mA cm^{-2}, which produced CO, H_2 and formate with FE of 75.7%, 21.2% and 75.5%, respectively. Therefore, the energy efficiency after 1 hour electrolysis was:

$$\varepsilon_{2.3\,V} = \frac{0.32}{2.3} \times 0.97 \times 0.76 \times 100\% = 10.2\%$$

Calculation of standard reduction potential for EGOR to formate

The reduction potential for EG/formic cid couple was calculated

$2HCO_2H + 6H^+ + 6e^- \rightarrow C_2H_6O_2 + 2H_2O$

ΔG^o_{rxn}=((ΔG^o_f(EG) + 2ΔG^o_f(H_2O) – 2ΔG^o_f(formate))

$$\Delta G^o_{rxn} = -394.3 - 2 \times 237.7 - 2 \times (-361.4)\ kj\ mol^{-1} = 146.9\ kj\ mol^{-1}$$

E^o_{rxn} *(vs RHE)* = $\Delta G^o_{rxn}/nF$

E^o_{rxn} (EG/formate) = 0.254 V

Supplementary Figures

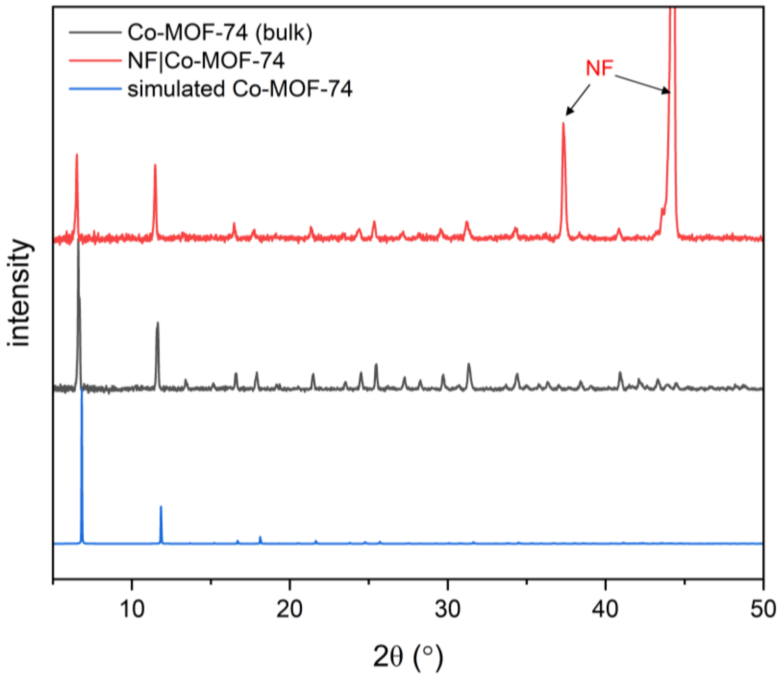

Figure S1. Powder X-ray diffraction (PXRD) pattern of bulk Co-MOF-74 powder and NF|Co-MOF-74 film compared with the simulated pattern based on the crystal structure of Co-MOF-74.

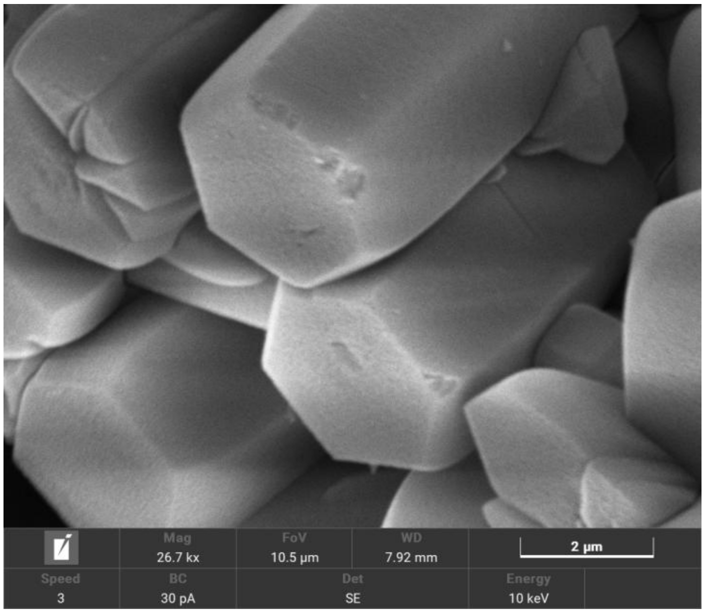

Figure S2. High-magnification SEM image of pristine Co-MOF-74.

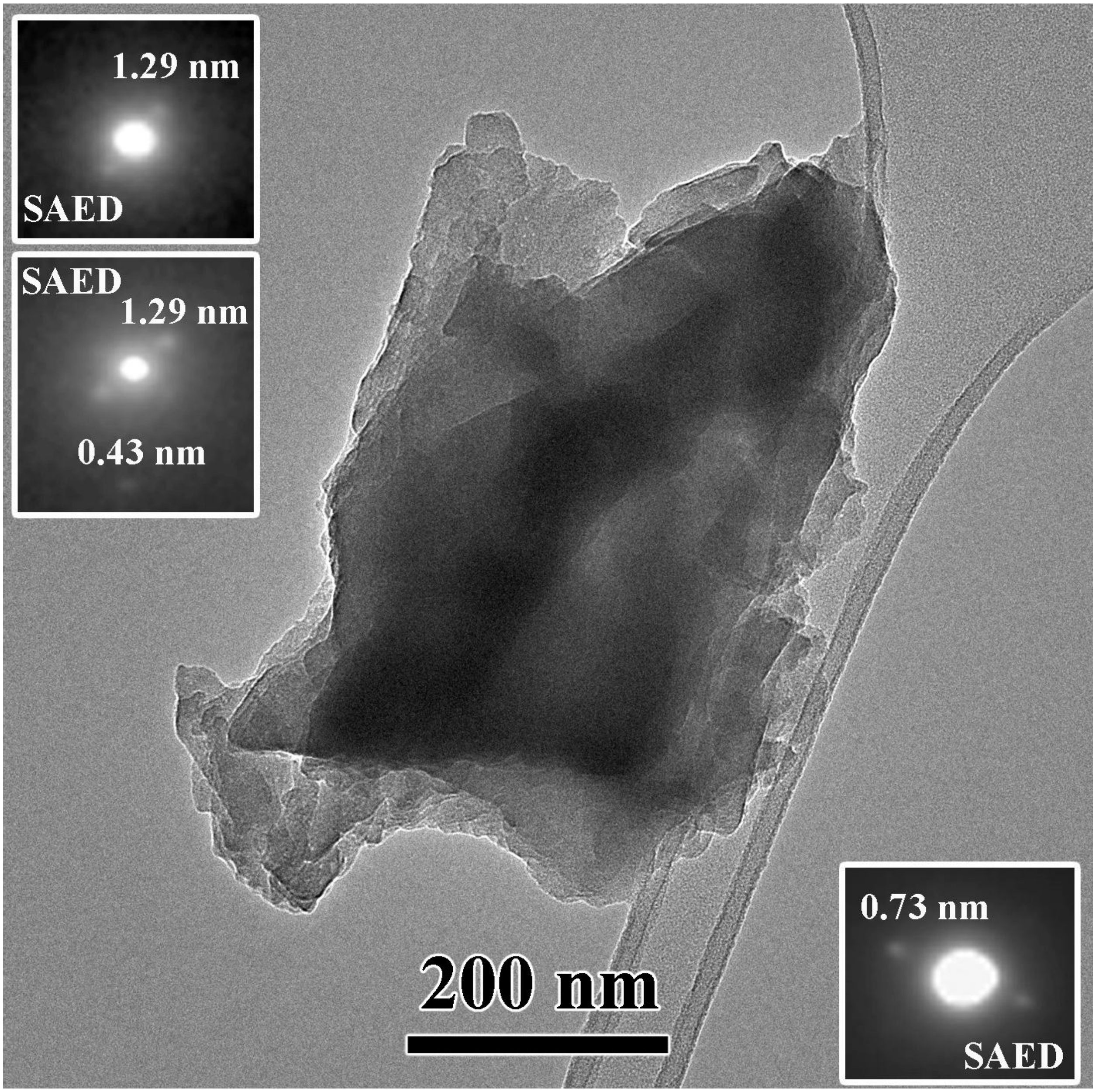

Figure S3. TEM image of surface-grown Co-MOF-74 including SAED patterns (insets)

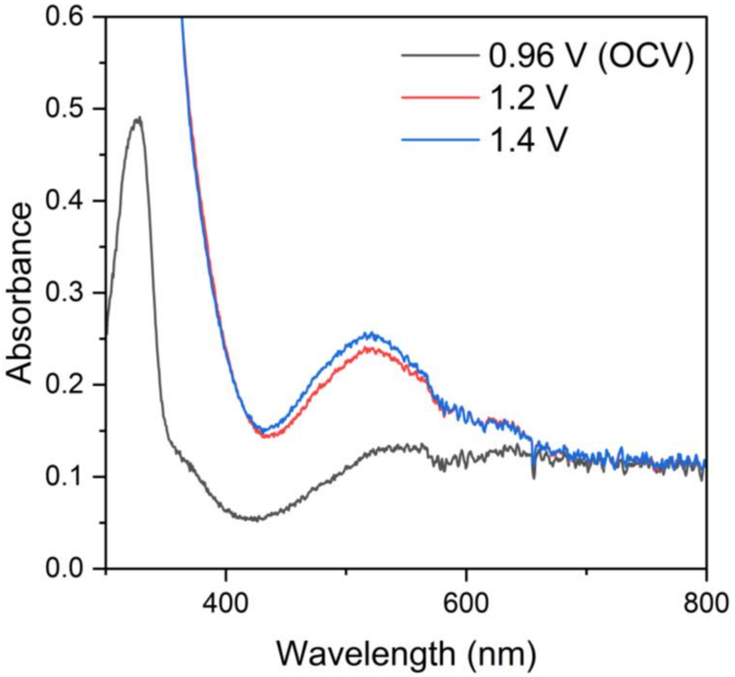

Figure S4. In situ UV-vis spectroelectrochemistry of the electrolyte during chronopotentiometry with a Co-MOF-74 electrode at different applied potential (vs. RHE). The catalyst loading on a carbon paper electrode was ~1 mg cm^{-2}. The electrode was held at each potential for 2 minutes with stirring before recording the spectrum. The black trace shows the pre-electrolysis UV-vis spectra (at open circuit voltage, OCV) of the electrolyte after equilibrating the electrode for 5 min.

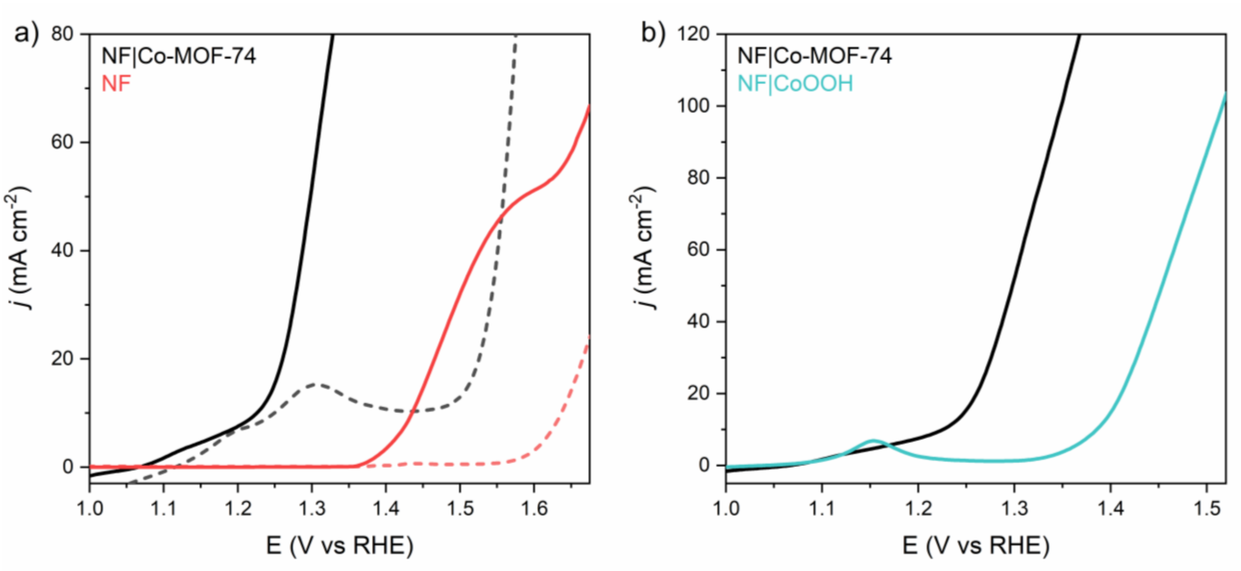

Figure S5. Comparison of linear sweep voltammograms: (a) blank NF (red lines) and NF|Co-MOF-74 (black lines) recorded in 1 M KOH in the presence (solid lines) and absence (dashed lines) of ethylene glycol (0.1 M), and (b) NF|Co-MOF-74 (black line) and NF|$Co(OH)_2$ (cyan line) recorded in 1 M KOH in the presence of ethylene glycol (0.1 M). Linear sweep voltammograms were recorded at a scan rate of 1 mV s^{-1} (90% iR-compensation).

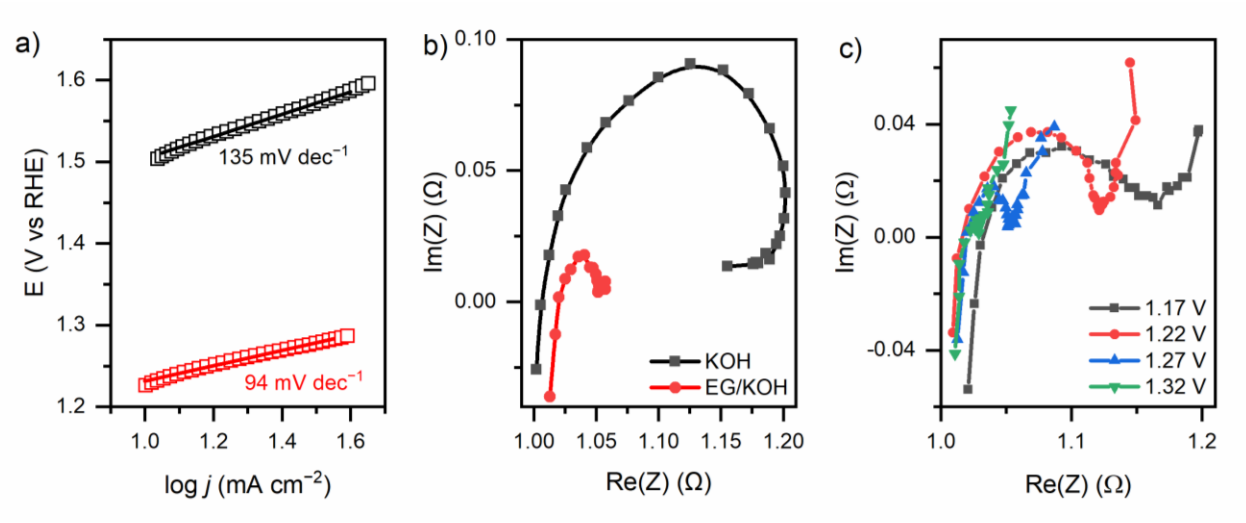

Figure S6. (a) Tafel plots and (B) Nyquist plots at the potential of 1.27 V of NF|Co-MOF-74 electrode in 1 M KOH electrolyte with and without 0.1 M EG. (C) Nyquist plots of NF|Co-MOF-74 electrode at different applied potential in 0.1 M EG in 1 M KOH.

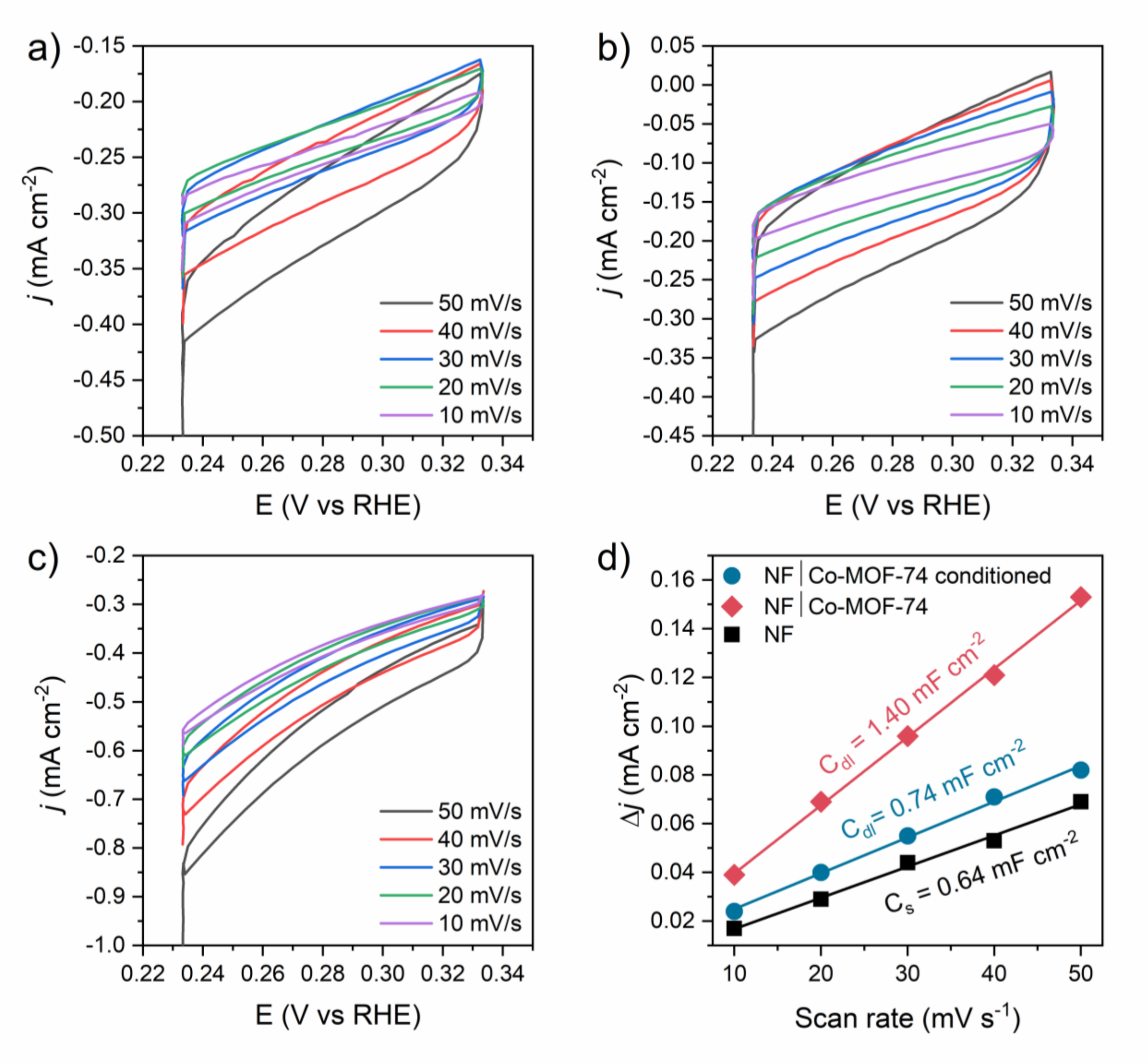

Figure S7. CV curves in the non-faradaic region from non-faradaic region (-0.234 to -0.334 V vs RHE) for (a) NF, (b) NF|Co-MOF-74, and (c) NF|Co-MOF-74 after 50 conditioning CV scans. (d) Linear fits of the increment of current density versus the scan rate for NF (dark), NF|Co-MOF-74 (red) and conditioned NF|Co-MOF-74 (blue) in the non-Faradaic region. Capacitive current densities were calculated from the difference between cathodic and anodic current densities at –0.284 V.

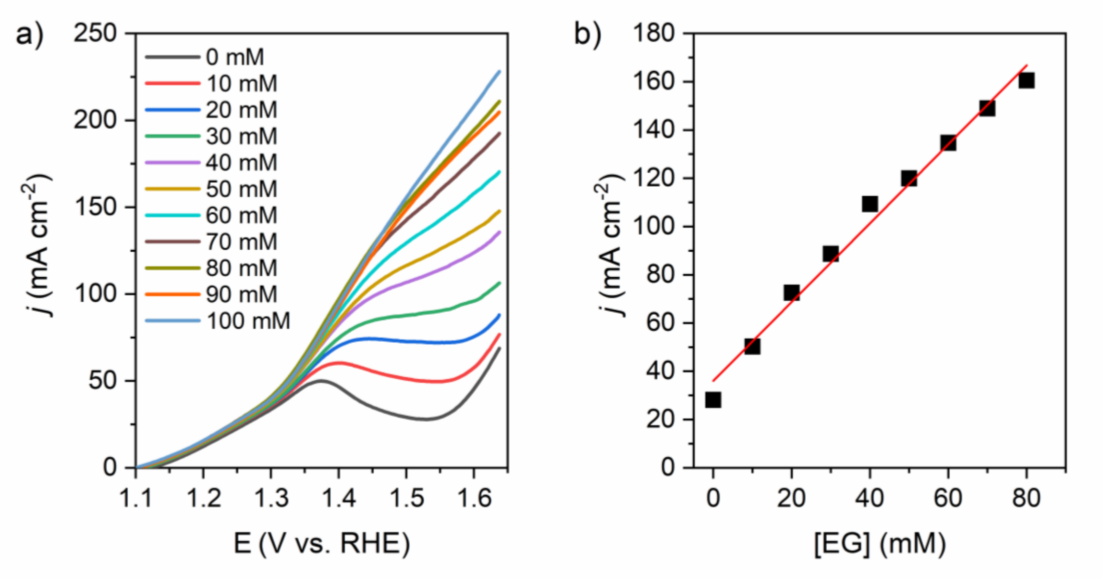

Figure S8. (a) LSV recorded for EGOR at different concentration using NF|Co-MOF-74 at 20mV s^{-1} scan rate. (b) Linear fit of the current density at 1.5 V vs EG concentration plot ($R^2 = 0.987$).

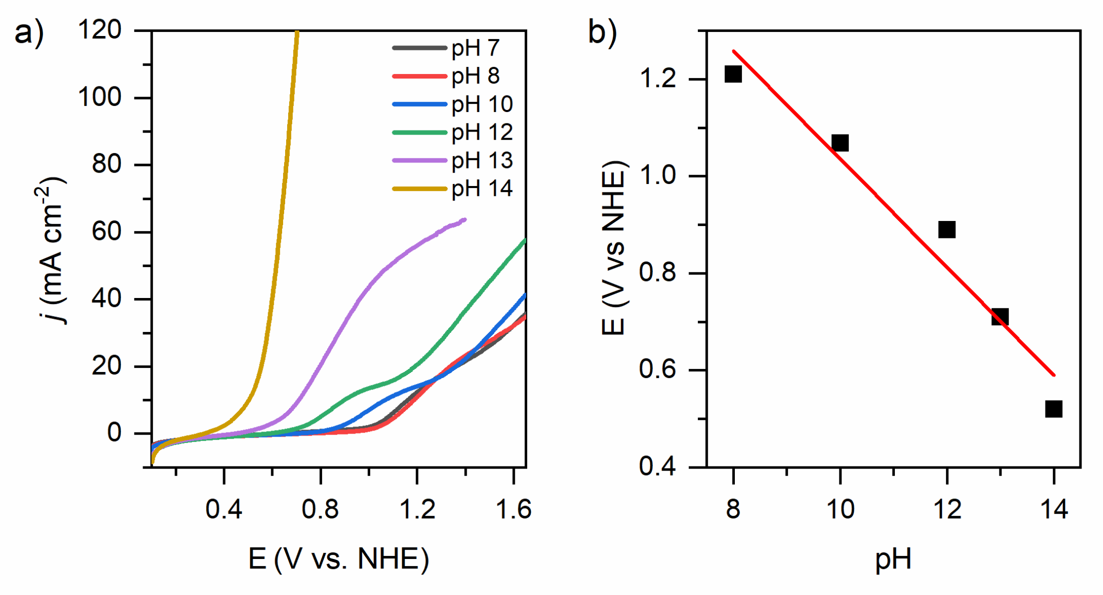

Figure S9. (a) LSV recorded for EGOR at different pH using NF|Co-MOF-74 at 20mV s^{-1} scan rate. Buffered electrolytes: phosphate buffer (0.1 M) adjusted to pH 7, 8 and 12; carbonate buffer (0.1 M) adjusted to pH 10, and KOH 0.1 M and 1 M for pH 13 and 14, respectively. (b) plot of E at 10 mA cm^{-2} versus pH for EGOR. The slope of the linear fit was 111 mV pH dec^{-1} with an R^2 value of 0.96.

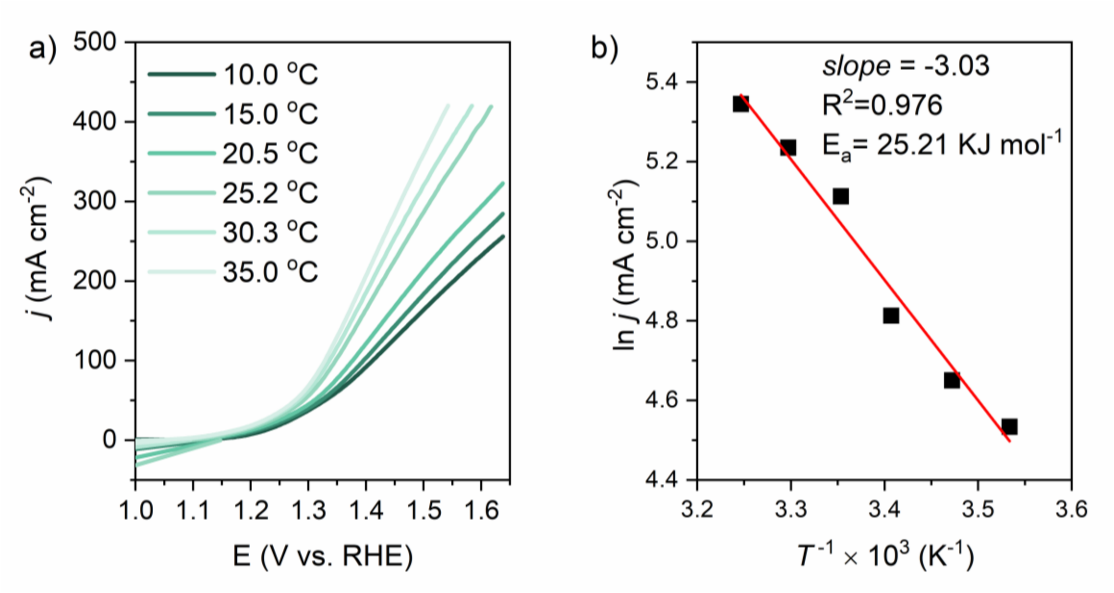

Figure S10. (a) LSV recorded for EGOR at different temperatures using NF|Co-MOF-74 at 20mV s^{-1} scan rate. (b) Arrhenius plot for log *j* (current density) versus 1/T at 1.4 V.

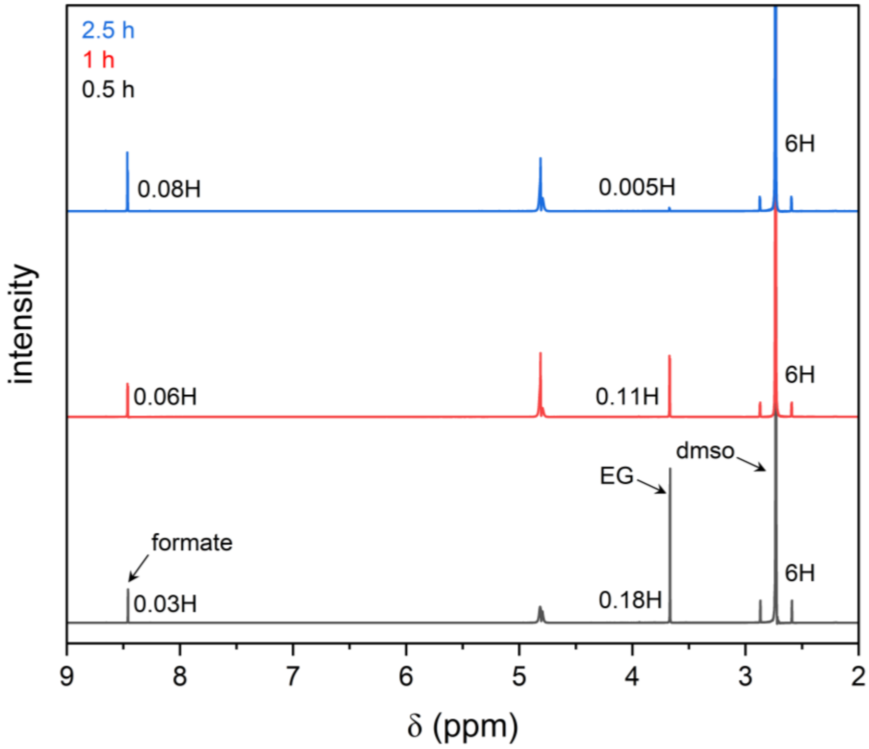

Figure S11. ^{1}H NMR of the electrolyte after 4-hour electrolysis at 1.40 V vs RHE. The CPE was performed with NF|Co-MOF-74 in 0.1 M EG in 1 M KOH. The NMR sample was prepared by mixing 0.05 mL electrolyte with 0.6 mL D_2O and 0.005 mL DMSO (71 µmol) as internal standard. The integration for the peaks is shown next to them. After 2.5 h CPE, the remaining amount of EG was ~26 µmol in 15 mL electrolyte.

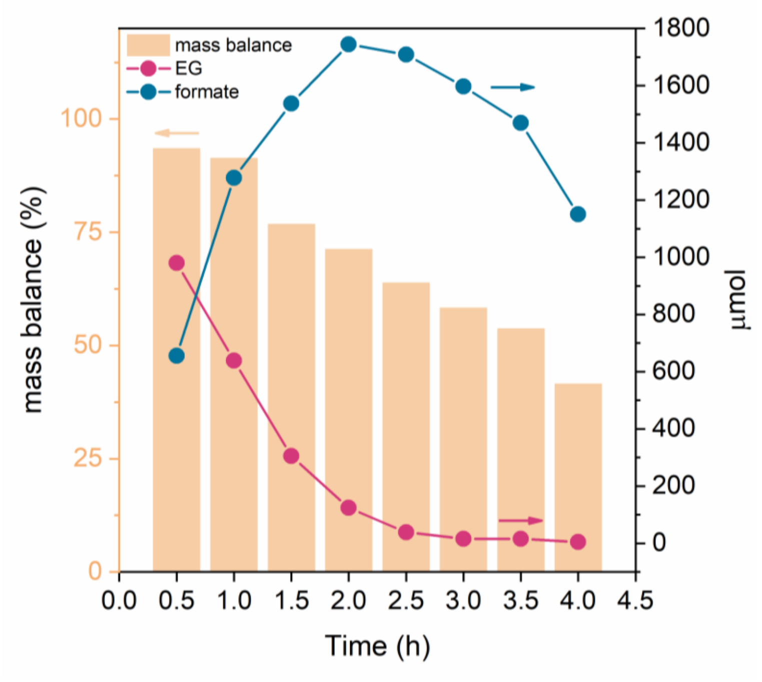

Figure S12. Mass balance of the EGOR of a 0.1 M EG solution in 1 M KOH, showing the evolution of EG and formate concentration over reaction time.

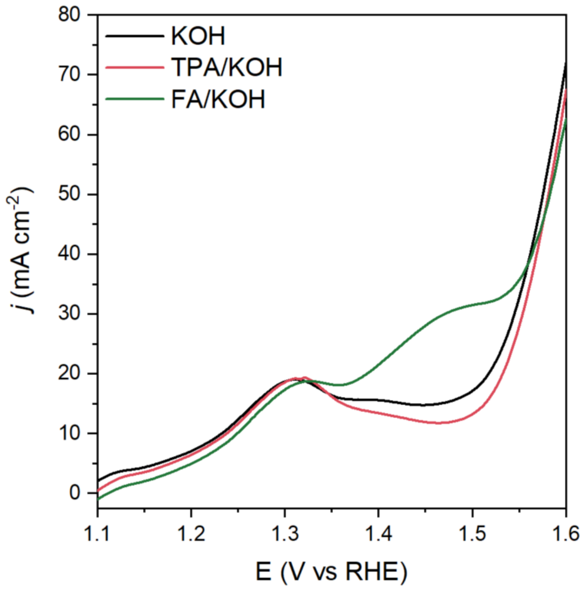

Figure S13. Linear sweep voltammetry of NF|Co-MOF-74 recorded in 1 M KOH in the presence of 0.1 M formate and 0.1 M terephthalic acid (TPA) at a scan rate of 1 mV s^{-1}.

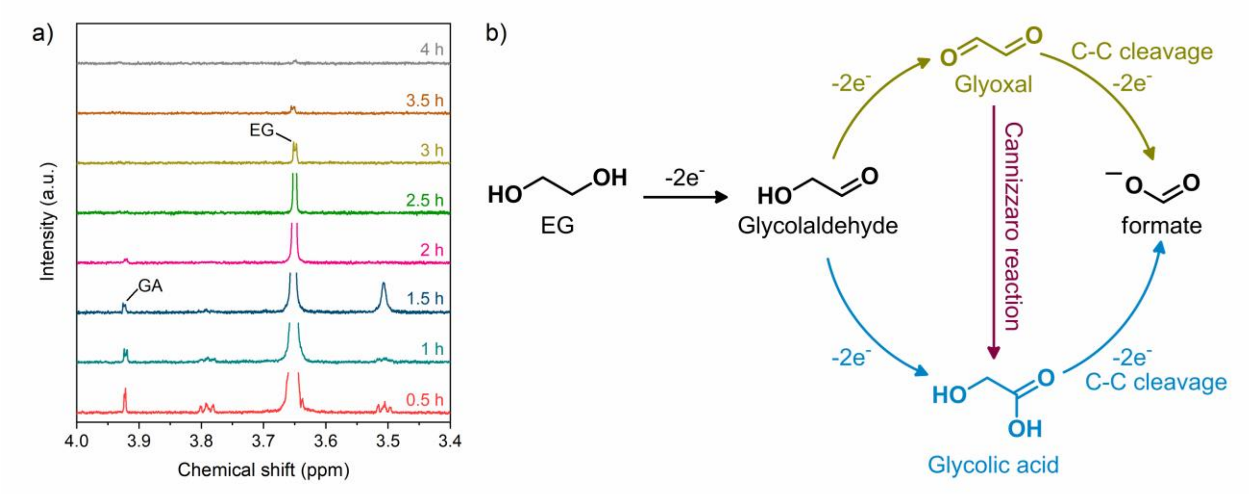

Figure S14. (a) ^{1}H NMR of the electrolyte showing formation of glycolic acid (GA). The electrolysis conditions are described in Figure S8 caption. (b) Proposed reaction pathways for EGOR to formate.

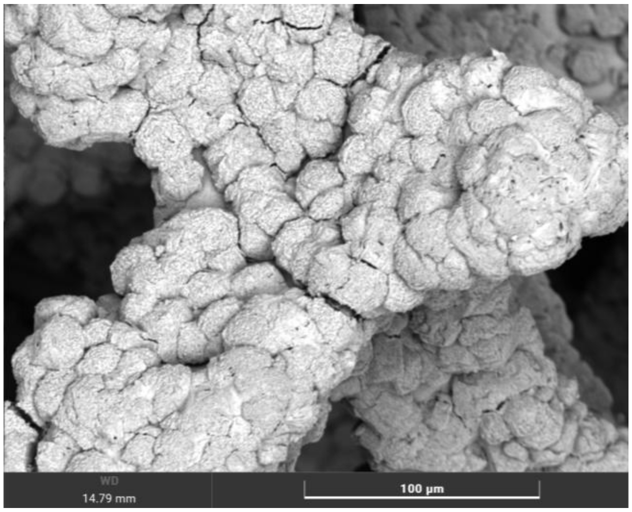

Figure S15. SEM image of post-catalysis NF|Co-MOF-74 recorded using a BSE detector.

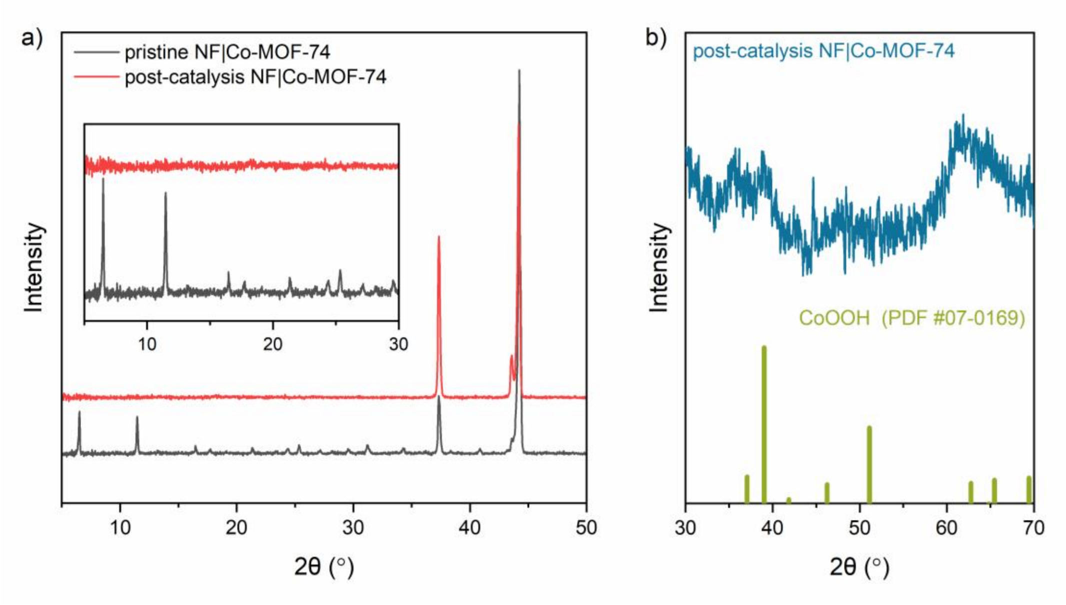

Figure S16. Powder X-ray-diffraction patterns of (a) pristine and post-catalysis NF|Co-MOF-74 and (b) detached Co-MOF-74 powder after catalysis.

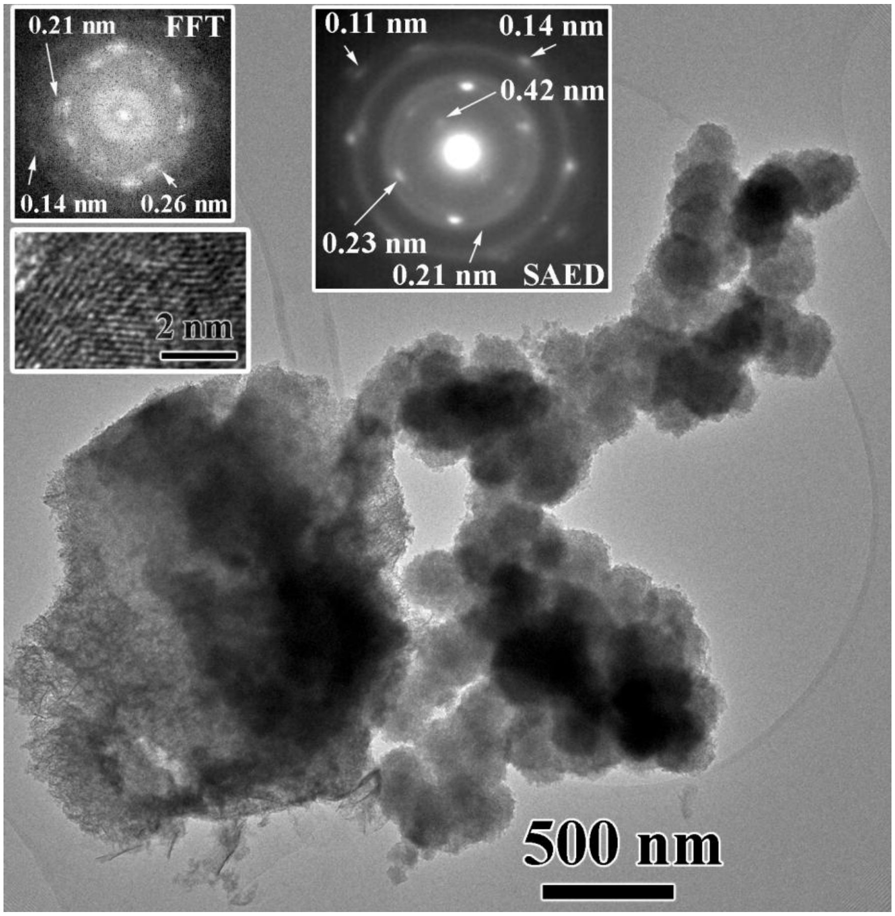

Figure S17. TEM image of post-catalysis Co-MOF-74 including SAED pattern. The upper left insets show HRTEM image and its corresponding FFT pattern.

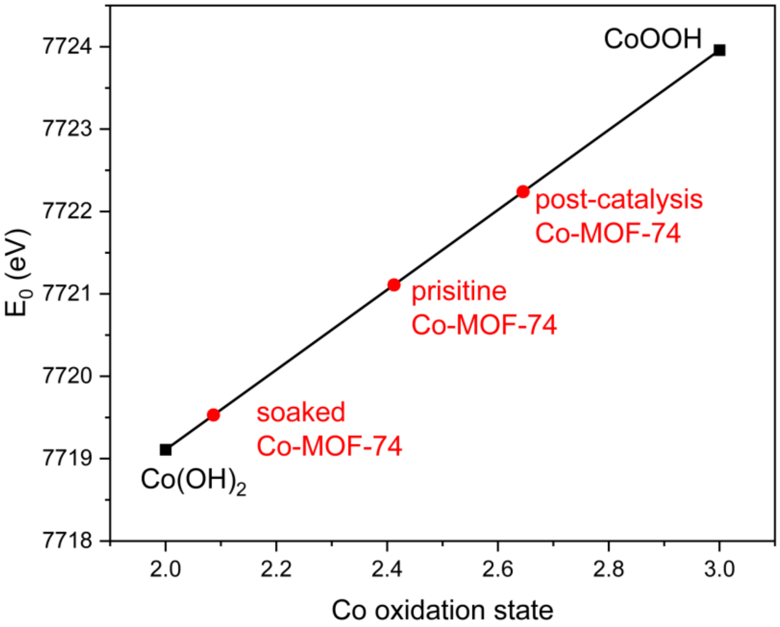

Figure S18. Plot of XANES edge energy versus the oxidation state of two standard Co samples and the corresponding linear fit (black trace). The E_0 values for pristine, electrolyte soaked, and post-catalysis Co-MOF-74 samples are shown in red.

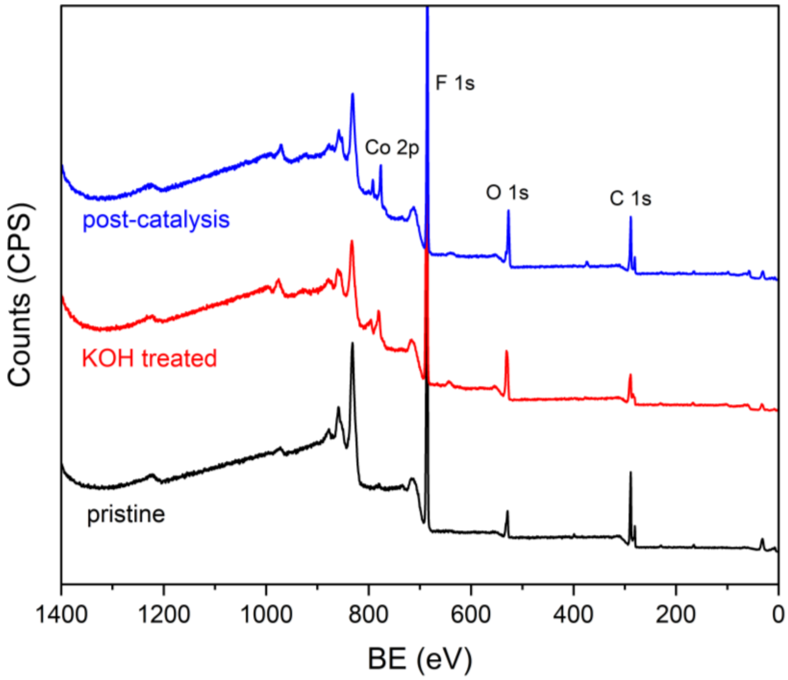

Figure S19. Survey XPS of a pristine CP|CoPPc electrode, after KOH treatment, and after electrolysis at 1.4 V vs RHE.

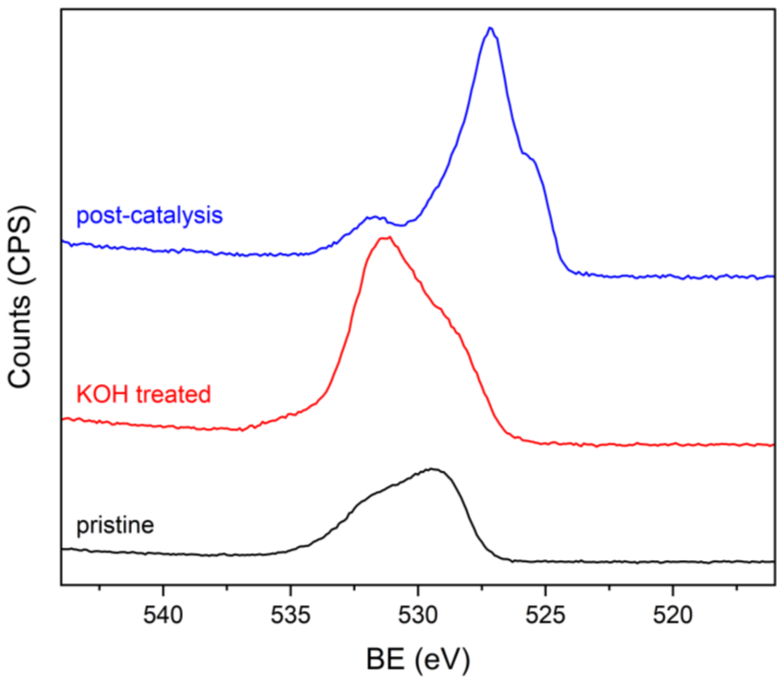

Figure S20. O 1s XPS of a pristine NF|Co-MOF-74 electrode, after KOH treatment, and after electrolysis at 1.4 V vs RHE in EG/KOH.

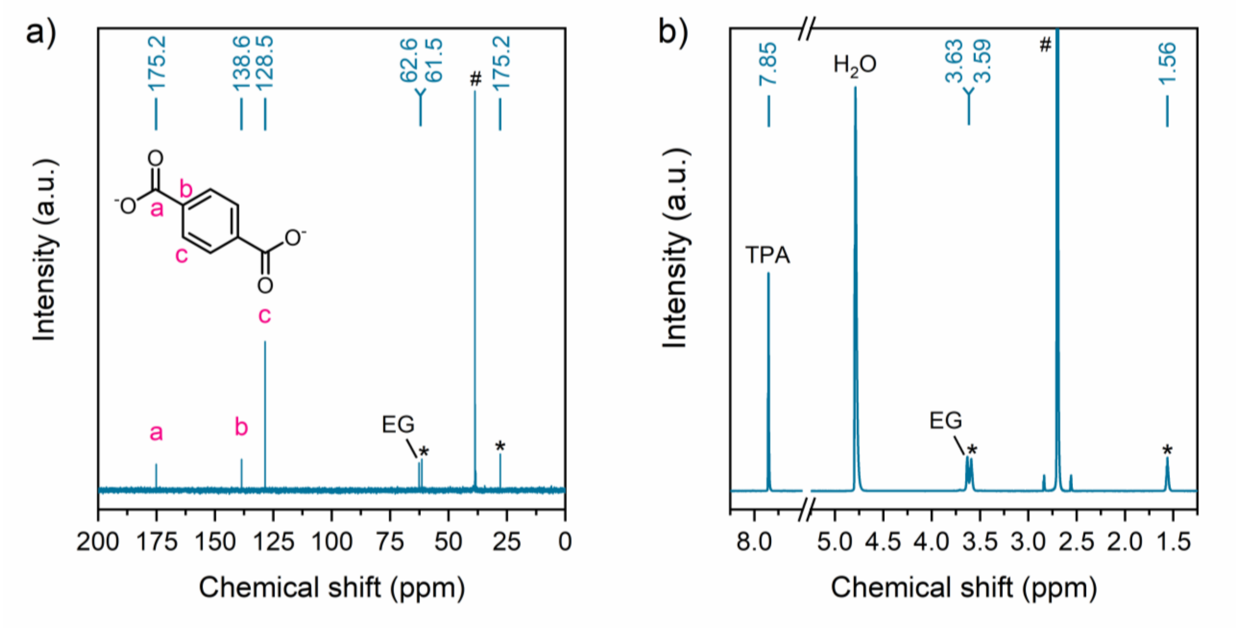

Figure S21. ^{1}H NMR (a) and ^{13}C NMR (b) of the PET hydrolysate solution after 48 h reaction. Hash and asterisk symbols denote signals from the internal standard (DMSO), and 1,4-butanediol, respectively.

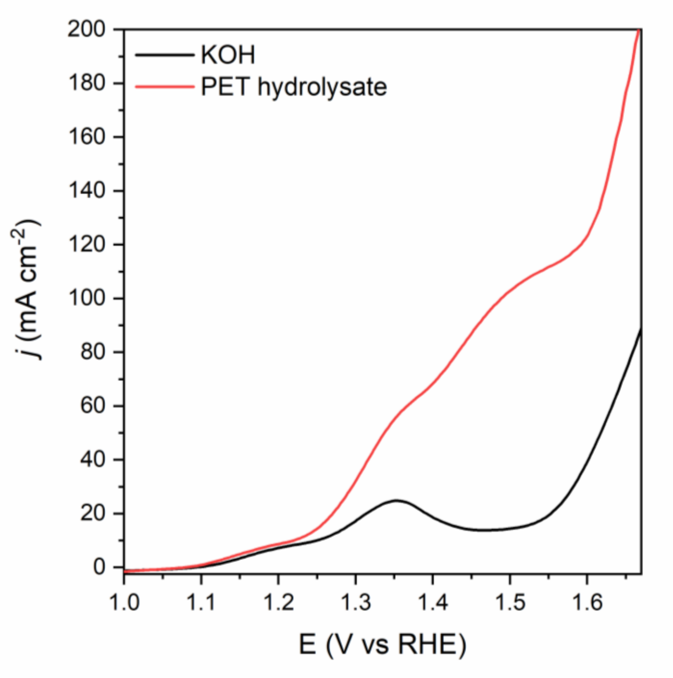

Figure S22. Linear sweep voltammetry of NF|Co-MOF-74 recorded in 1 M KOH and using PET hydrolysate (2 M KOH and 24 mM EG) at a scan rate of 1 mV s^{-1}.

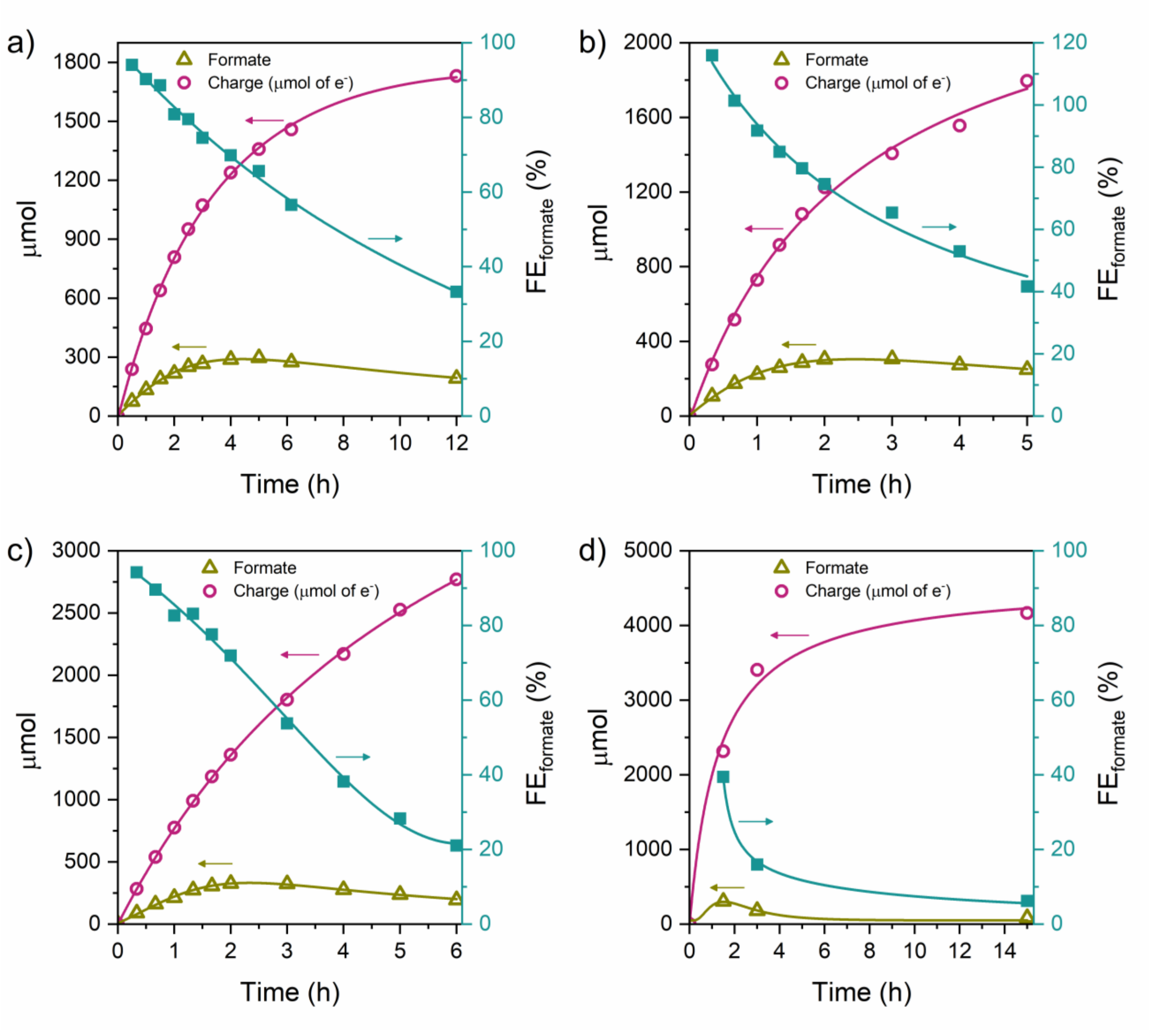

Figure S23. Ethylene glycol oxidation reaction (EGOR) mediated by NF|Co-MOF-74 at different applied potentials: (a) 1.29 V vs RHE, (b) 1.33 V vs RHE, (c) 1.39 V vs RHE, and (d) and 1.43 V vs RHE. The purple and dark yellow traces show the charge passed and amount of formate, respectively. The cyan trace shows the FE for formate.

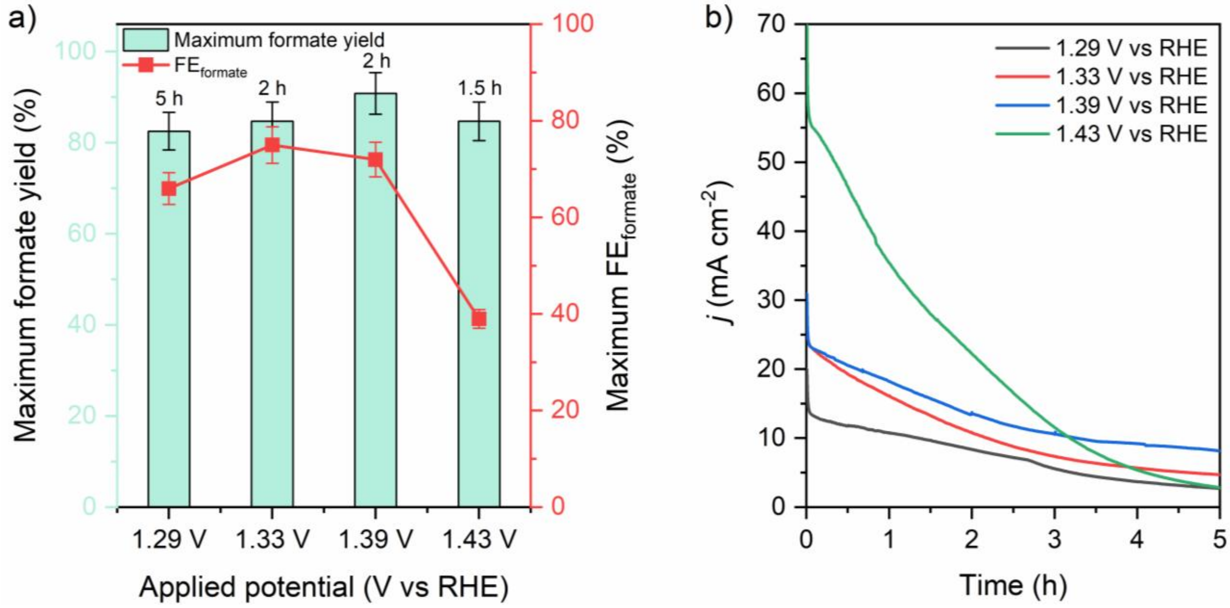

Figure S24. (a) Comparative EG conversion to formate and Faradaic Efficiency to formate ($FE_{formate}$) for NF|Co-MOF-74 electrode at different applied potentials. The maximum formate yield was calculated using the equation, $Yield\ (\%) = \frac{formate_{\mu mol}}{EG_{\mu mol} \times 2} \times 100$. 7.5 mL hydrolysate containing 25 mM EG was used for electrolysis. (b) The chronoamperometry EGOR curve of NF|Co-MOF-74 electrode using PET hydrolysate (24 mM EG) as electrolyte at applied potentials ranging from 1.29 to 1.43 V vs RHE.

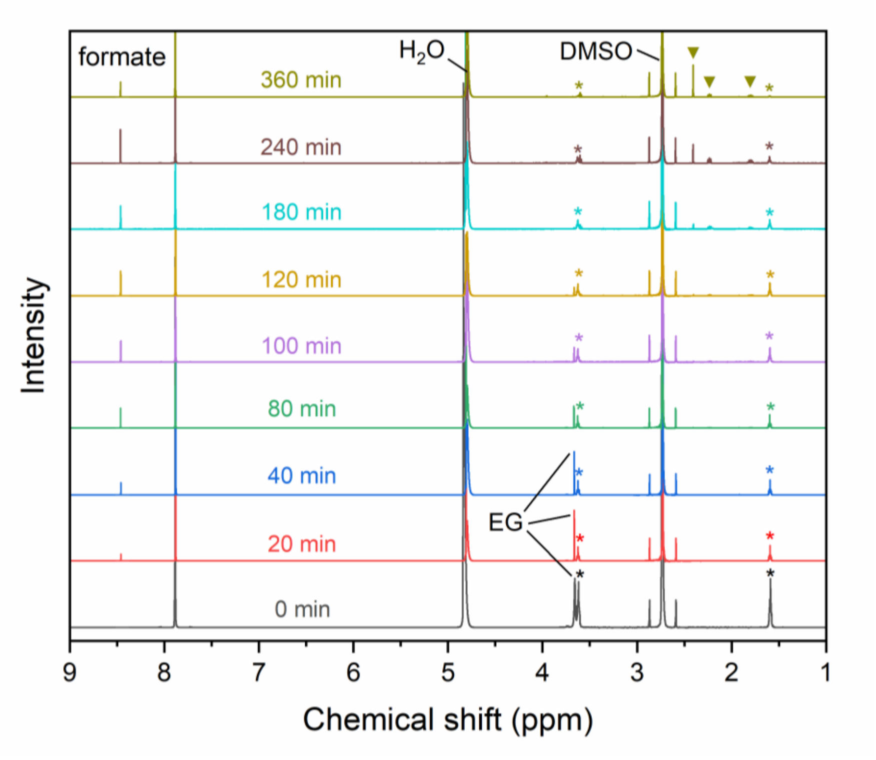

Figure S25. ^{1}H NMR of PET hydrolysate during electrolysis using NF|Co-MOF-74 at an applied potential of 1.39 V vs RHE. The peaks corresponding to 1,4-butanediol are marked with asterisk. The triangle symbols mark the oxidation product from 1,4-butanediol.

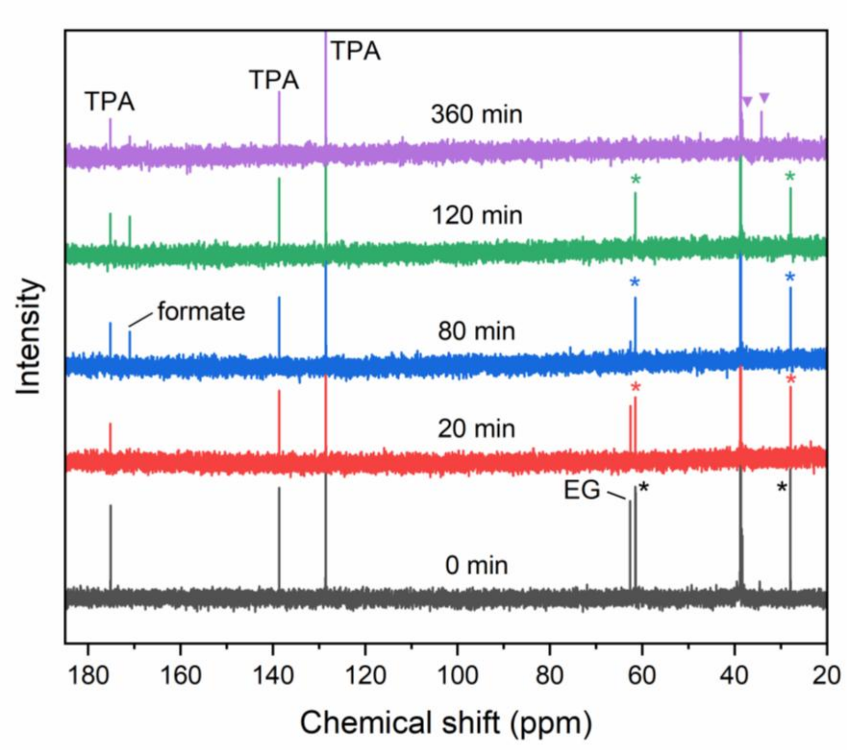

Figure S26. ^{13}C NMR of PET hydrolysate during electrolysis using NF|Co-MOF-74 at an applied potential of 1.39 V vs RHE. The peaks corresponding to 1,4-butanediol are marked with asterisk. The triangle symbols mark the oxidation product from 1,4-butanediol. The peaks corresponding to terephthalic acid are marked with TPA.

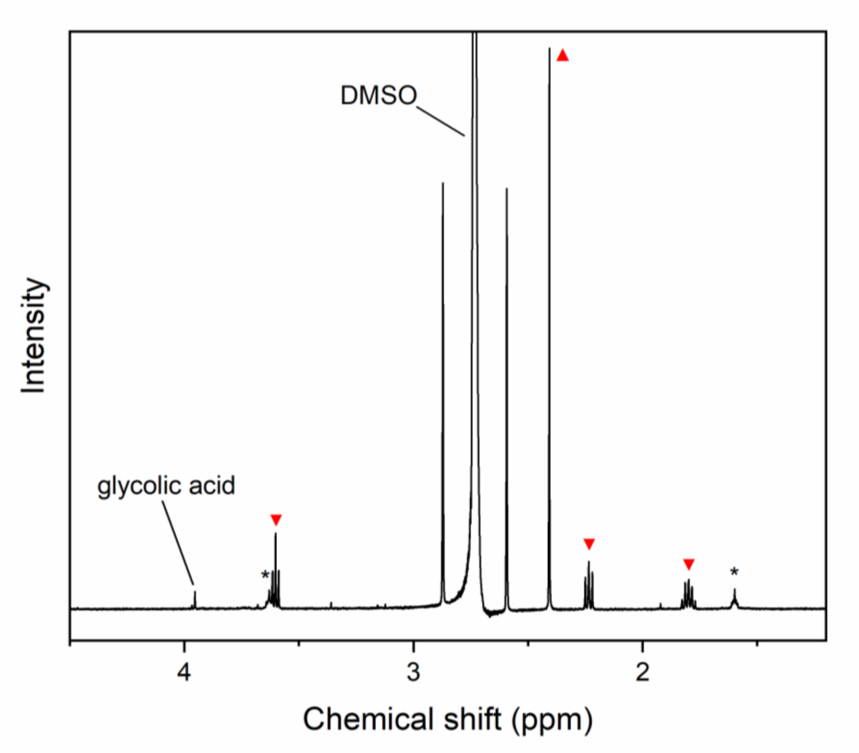

Figure S27. ^{1}H NMR of PET hydrolysate after 6 h electrolysis at 1.39 V vs RHE using a NF|Co-MOF-74 electrode. The peaks corresponding to 1,4-butanediol are marked with asterisk. The red triangle symbols mark the new peaks from the electrooxidation side reaction.

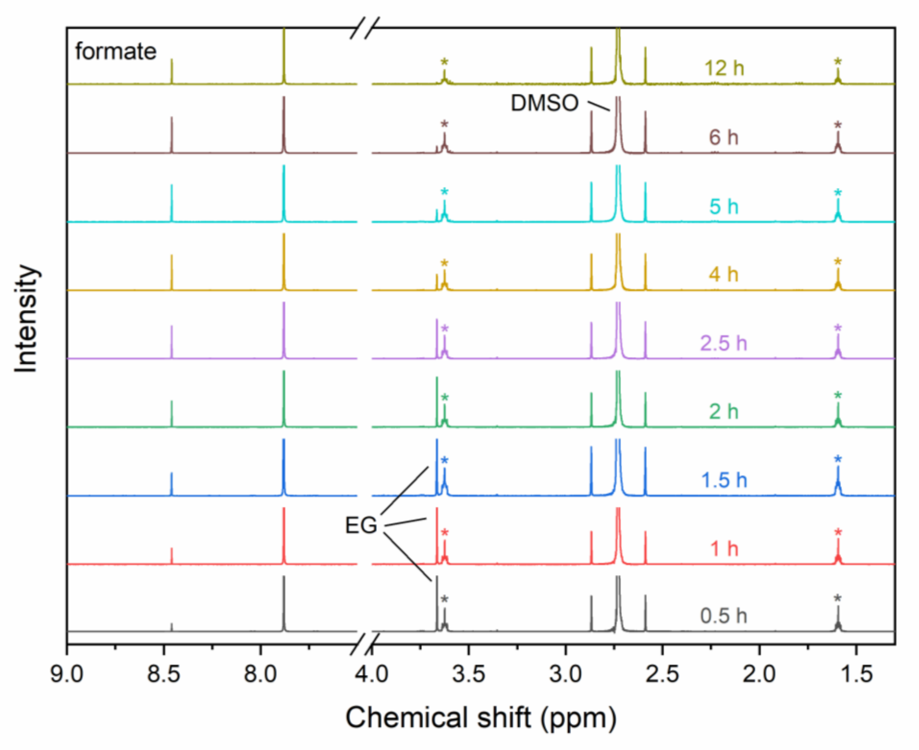

Figure S28. ^1H NMR of PET hydrolysate during electrolysis using NF|Co-MOF-74 at an applied potential of 1.29 V vs RHE. The peaks corresponding to 1,4-butanediol are marked with asterisk.

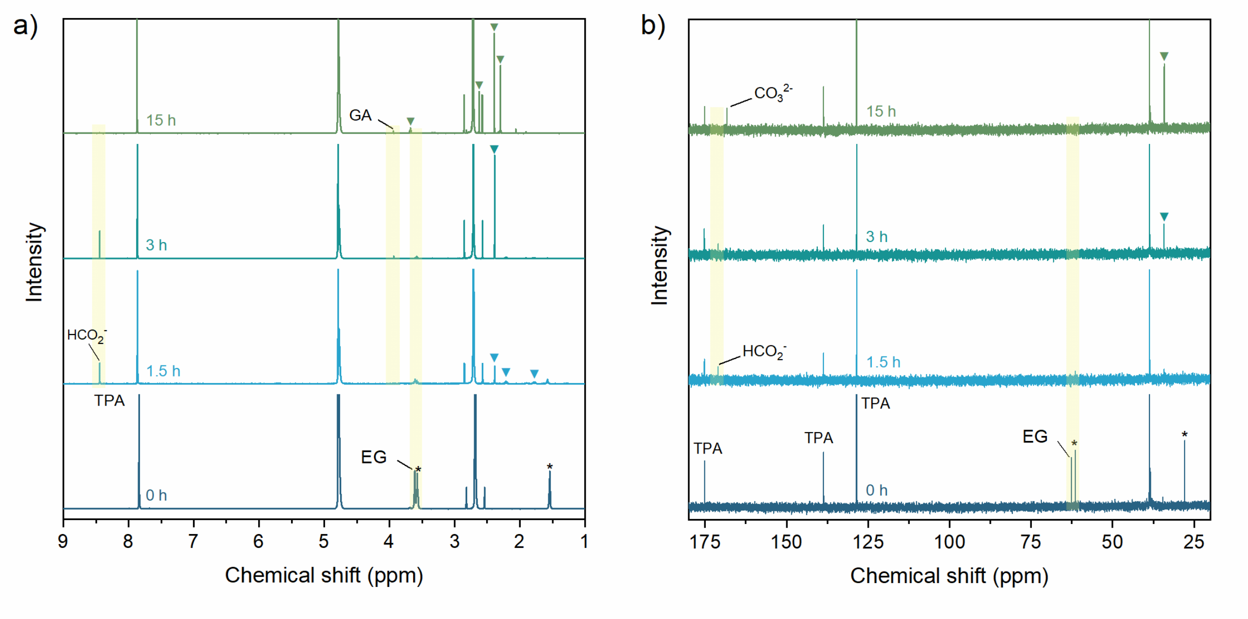

Figure S29. ^{1}H NMR (a) and ^{13}C NMR (b) of PET hydrolysate during electrolysis using NF|Co-MOF-74 at an applied potential of 1.43 V vs RHE.

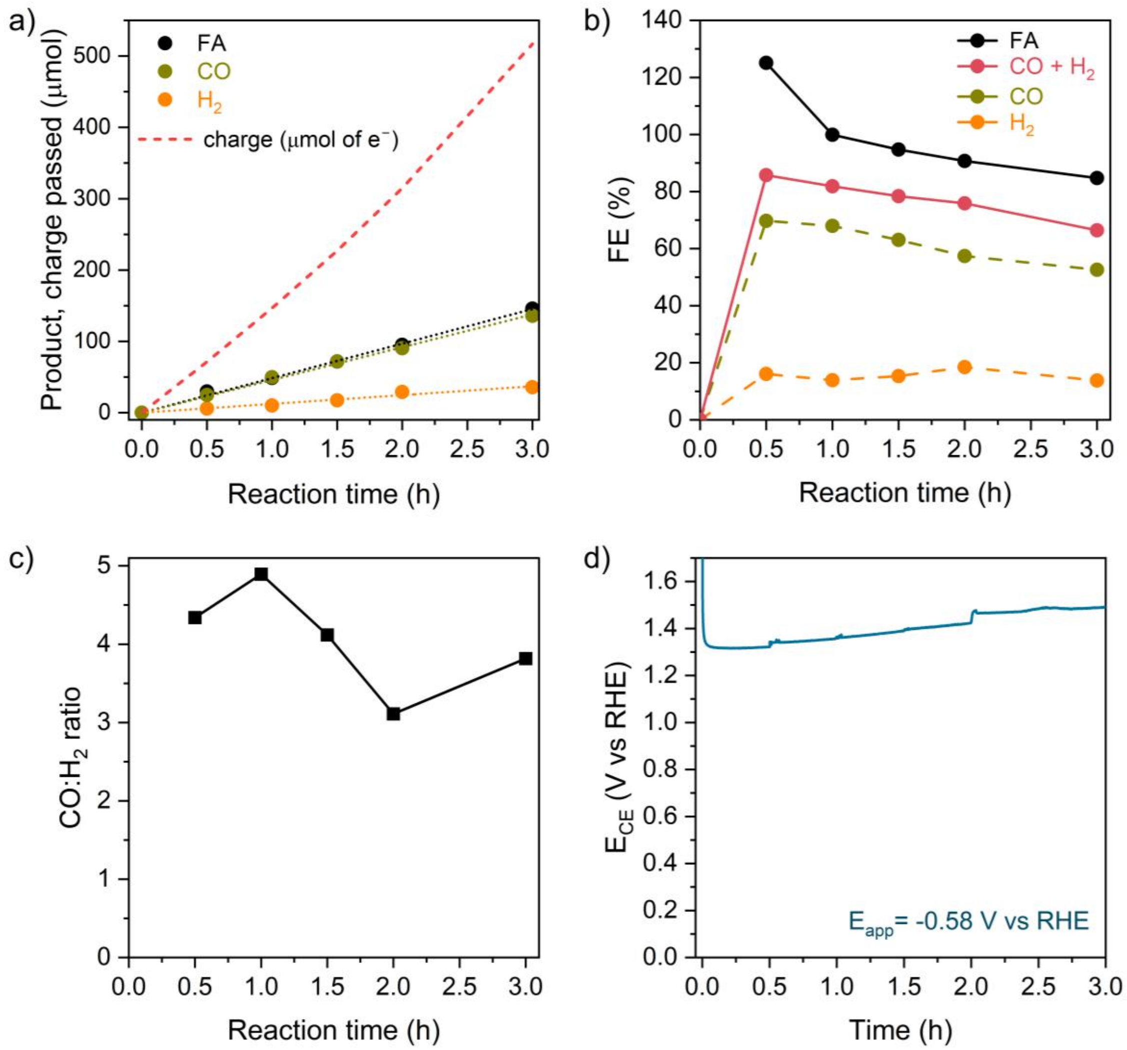

Figure S30. Coupled electrolysis data for CO_2R and EGOR performed in a 3-electrode configuration using CP|CoPPc as the cathode and NF|Co-MOF-74 as the anode ($E_{WE(CP|CoPPc)}$ = –0.58 V vs RHE). (a) Quantification of products (liquid and gaseous) and charge passed (dashed red trace, as recorded by the potentiostat, where 1 mol of e^-= 96,485 C or 1 Faraday) are shown against the reaction time. (b) Faradaic efficiency of formate, CO and H_2 against the reaction time. Total FE for syngas (CO + H_2) is shown as the red trace. (c) Variation of CO:H_2 ratio over 3 h coupled electrolysis. (d) Trend of the CE (counter electrode, NF|Co-MOF-74) potential over the reaction time.

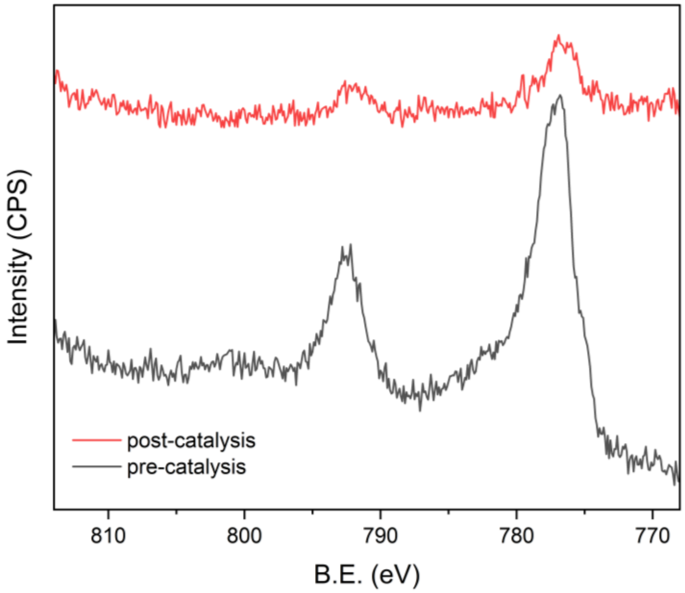

Figure S31. Co 2p XPS of a CP|CoPPc electrode before and after electrolysis at –0.58 V vs RHE in CO_2 saturated $NaHCO_3$ (0.5 M).

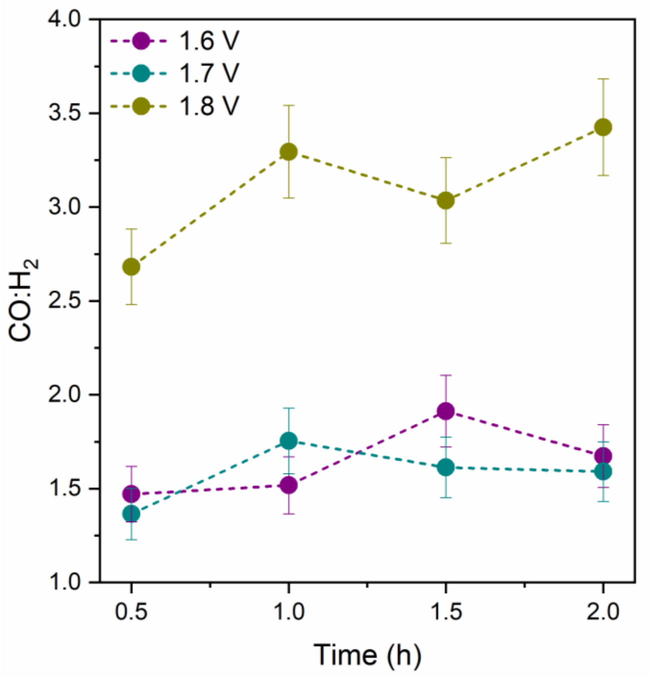

Figure S32 Variation of $CO:H_2$ ratio over 2 h coupled electrolysis in a 2-electrode configuration using PET hydrolysate as the anolyte. CP|CoPPc was used as the cathode and NF|Co-MOF-74 as the anode.

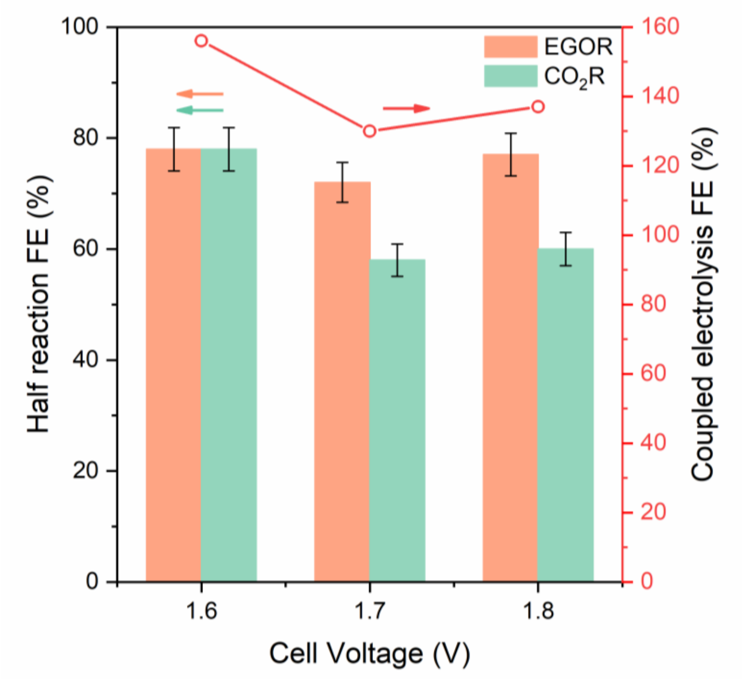

Figure S33. Paired electrolysis data showing the FE for EGOR from PET hydrolysate and CO_2 reduction. The left side axis shows the FE for half reactions ($FE_{formate}$ for EGOR from PET hydrolysate, and FE_{CO+H2} for CO_2R). The right-side axis presents the combined FE for anodic and cathodic reactions.

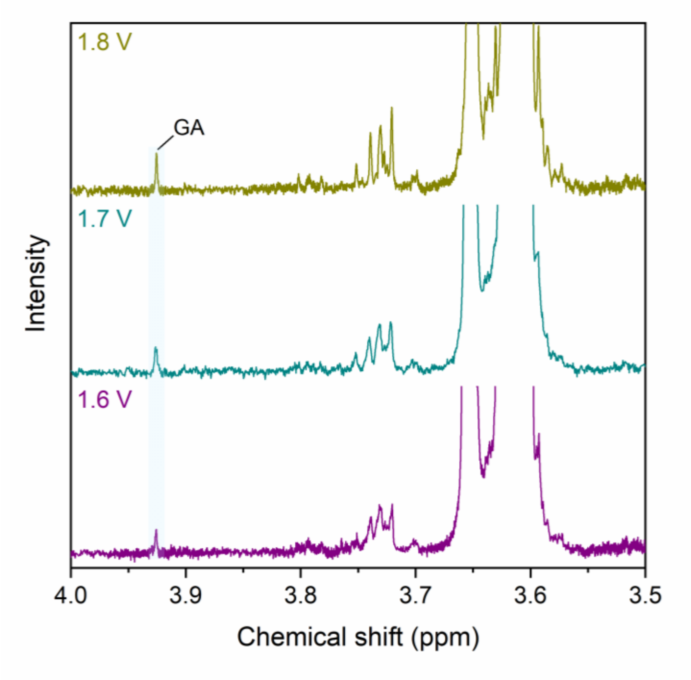

Figure S34. ^{1}H NMR of PET hydrolysate after 2h electrolysis showing the presence of glycolic acid (3.94 ppm) in the final anolyte.

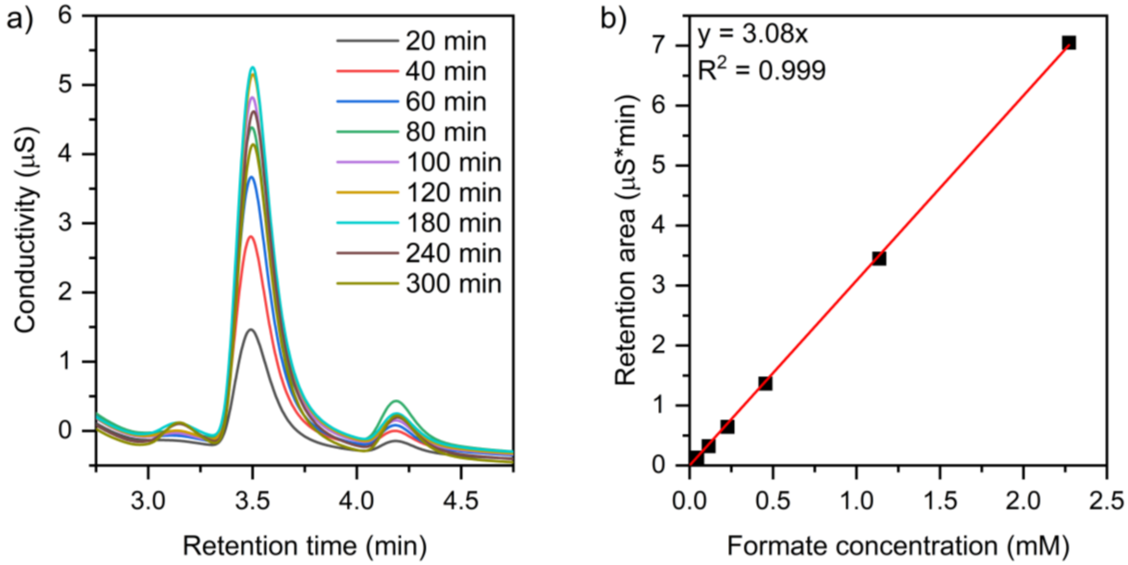

Figure S35. (a) Representative ionic chromatograms (IC) showing the magnified formate region of the EGOR electrolysate at a constant potential of 1.33 V vs. RHE. (b) Ionic chromatography calibration curve for formate concentration.

Supplementary Tables

Table S1. Atomic percentages from EDS for pre- and post-catalysis NF|Co-MOF-74

Element	Pre-catalysis NF|Co-MOF-74	Post-catalysis NF|Co-MOF-74
C	53.13%	28.01%
O	31.98%	40.62%
Co	9.23%	20.18%
Ni	3.75%	5.4%

Table S2. Comparative performances of different catalysts for EGOR in alkaline medium.

Electrocatalyst	Current density (mA/cm^2)	Potential (vs RHE)	Electrolyte	Ref.
NF|$CoNi_{0.25}P$	50	1.31	1 M KOH + 0.3 M EG	[3]
NF|Ni–Co_9S_8 NSAs	100	1.28	1 M KOH + 1 M EG	[4]
CF|CuO NWs	10	1.38	1 M KOH + 0.1 M EG	[5]
NF|$CuCo_2O_4$ NWA	100	1.48	1 M KOH + 0.4M EG[(a)]	[6]
CFP|$NiCo_2O_4$	50	1.45	1 M NaOH + 0.1 M EG[(a)]	[7]
CP|CuCoO@rGO	20	1.45	1 M KOH + 42 mM[(a)]	[8]
CF|CuO@$Ni(OH)_2$	50	1.36	1 M KOH + 0.1 M EG	[9]
NF|Mn/CoOOH	100	1.30	1 M KOH + 0.3 M EG	[10]
NF|$Ni(OH)_2$-V_O	100	1.43	1 M KOH + 0.2 M EG[(a)]	[11]
NF| NiMOF@MnCo-OH	10	1.34	1 M KOH + 0.1 M EG	[12]
NF| CoV-LDH	150	1.38	1 M KOH + 0.15 M EG	[13]
NF|Ni_3S_2@NiFeMn-LDH	100	1.42	1 M KOH + 1 M EG	[14]
NF|NiFe-LDH	100	1.52	1 M KOH + 0.5 M EG	[15]
NF|Co-MOF-74	100	1.35	1 M KOH + 0.1 M EG	*This work*

Abbreviations: NF = nickel foam; CF = copper foam; CFP = carbon fiber paper; CP = carbon paper. [(a)]Concentration of EG in PET hydrolysate

Table S3. Comparison of the cell energy efficiency of previous reported coupled AlcOx-CO_2R electrolysers.

Catalysts	Overall reaction	V_{cell} (V)	ε (%)	Ref.
$Ni(OH)_2$-NF (AlcOx) Biene(BDC) (CO_2R)	$CH_3OH + 2CO_2 + H_2O \rightarrow 3HCO_2H$	2<br>2.2<br>2.4	14.1[(a)]<br>12.8[(a)]<br>11.8[(a)]	16
CuONS/CF (AlcOx) $mSnO_2$/CC (CO_2R)	$CH_3OH + 2CO_2 + H_2O \rightarrow 3HCO_2H$	1.22	16.9[(a)]	17
S-NiCo-LDH (AlcOx) $BiPO_4$ derived 2D nanosheets (CO_2R)	$CH_3OH + 2CO_2 + H_2O \rightarrow 3HCO_2H$	2.06<br>2.28	14.3[(a)]<br>10.8[(a)]	18
mesoITO|STEMPO (AlcOx) CoPPc (CO_2R)	$2C_3H_8O_3 + CO_2 \rightarrow 2C_3H_6O_3 + CO + H_2 + H_2O$	1.8<br>1.9<br>2	17.1[(b)]<br>14.8[(b)]<br>13.3[(b)]	19
nano-ITO|[Ru(bis-Mebimpy)(4,4′-($(OH)_2OPCH_2)_2$-bpy)(OH_2)$]_2$ (AlcOx) [(tpy)(Mebim-py)RuII(OH_2)$]^{2+}$ (CO_2R)	$2PhCH_2OH + CO_2 \rightarrow 2PhCHO + CO + H_2 + H_2O$	1.8	6.7[(c)]	20
$NiCo_2O_4$/CFP (AlcOx) SnO_2/CC (CO_2R)	$C_2H_6O_2 + 3CO_2 + 2H_2O \rightarrow 5HCO_2H$	1.7<br>1.8<br>1.9	7.5[(d)]<br>9.2[(d)]<br>9.4[(d)]	7
CuCoO@rGO (AlcOx) BOC@rGO (CO_2R)	$C_2H_6O_2 + 3CO_2 + 2H_2O \rightarrow 5HCO_2H$	1.7<br>1.8<br>1.9	7.5[(d)]<br>8.1[(d)]<br>9.4[(d)]	8
NF|Co-MOF-74 (AlcOx) CoPPc (CO_2R)	$C_2H_6O_2 + 2CO_2 \rightarrow 2HCOOH + 2CO + H_2$	1.6<br>1.7<br>1.8<br>2.3	12.1[(e)]<br>7.9[(e)]<br>8.2[(e)]<br>10.2[(f)]	*This work*

[(a)] Cell energy efficiency (ε) values were calculated using $\Delta G^0_{rxn} = 109.2\ kJ\ mol^{-1}$ (n = 4 electrons); $E^0_{rxn} = 0.283\ V$

[(b)] Cell energy efficiency (ε) values were calculated using $\Delta G^0_{rxn} = 174.4\ kJ\ mol^{-1}$ (n = 4 electrons); $E^0_{rxn} = 0.45\ V$; FE_{CO}, FE_{H2} and $FE_{glyceraldehyde}$ values were estimated from the Figure in the reference.

[(c)] Cell energy efficiency (ε) values were calculated using $\Delta G^0_{rxn} = 174.4\ kJ\ mol^{-1}$ (n = 4 electrons); $E^0_{rxn} = 0.49\ V$ (FE_{PhCHO} ~70%, and FE_{CO+H2} ~35%)

[(d)] Cell energy efficiency (ε) values were calculated using $\Delta G^0_{rxn} = 189.2\ kJ\ mol^{-1}$ (n = 4 electrons); $E^0_{rxn} = 0.30\ V$; FE_{HCOOH} for cathodic and anodic reactions were estimated from the Figure in the reference.

[(e)] Chronoamperometry performed in H-cell

[(f)] Chronopotentiometry performed in flow-cell at 75 mA cm^{-2}

References

(1) Yang, J.; Liu, H.; Martens, W. N.; Frost, R. L. Synthesis and Characterization of Cobalt Hydroxide, Cobalt Oxyhydroxide, and Cobalt Oxide Nanodiscs. *J. Phys. Chem. C* **2010**, *114* (1), 111–119. https://doi.org/10.1021/jp908548f.

(2) Grim, R. G.; Huang, Z.; Guarnieri, M. T.; Ferrell, J. R.; Tao, L.; Schaidle, J. A. Transforming the Carbon Economy: Challenges and Opportunities in the Convergence of Low-Cost Electricity and Reductive CO_2 Utilization. *Energy Environ. Sci.* **2020**, *13* (2), 472–494. https://doi.org/10.1039/C9EE02410G.

(3) Zhou, H.; Ren, Y.; Li, Z.; Xu, M.; Wang, Y.; Ge, R.; Kong, X.; Zheng, L.; Duan, H. Electrocatalytic Upcycling of Polyethylene Terephthalate to Commodity Chemicals and H_2 Fuel. *Nat. Commun.* **2021**, *12* (1), 4679. https://doi.org/10.1038/s41467-021-25048-x.

(4) Ma, Y.; Li, L.; Tang, J.; Hu, Z.; Zhang, Y.; Ge, H.; Jian, N.; Zhao, J.; Cabot, A.; Li, J. Electrochemical PET Recycling to Formate through Ethylene Glycol Oxidation on Ni–Co–S Nanosheet Arrays. *J. Mater. Chem. A* **2024**, *12* (48), 33917–33925. https://doi.org/10.1039/D4TA07156E.

(5) Wang, J.; Li, X.; Zhang, T.; Chen, Y.; Wang, T.; Zhao, Y. Electro-Reforming Polyethylene Terephthalate Plastic to Co-Produce Valued Chemicals and Green Hydrogen. *J. Phys. Chem. Lett.* **2022**, *13* (2), 622–627. https://doi.org/10.1021/acs.jpclett.1c03658.

(6) Liu, F.; Gao, X.; Shi, R.; Tse, E. C. M.; Chen, Y. A General Electrochemical Strategy for Upcycling Polyester Plastics into Added-Value Chemicals by a $CuCo_2O_4$ Catalyst. *Green Chem.* **2022**, *24* (17), 6571–6577. https://doi.org/10.1039/D2GC02049A.

(7) Wang, J.; Li, X.; Wang, M.; Zhang, T.; Chai, X.; Lu, J.; Wang, T.; Zhao, Y.; Ma, D. Electrocatalytic Valorization of Poly(Ethylene Terephthalate) Plastic and CO_2 for Simultaneous Production of Formic Acid. *ACS Catal.* **2022**, *12* (11), 6722–6728. https://doi.org/10.1021/acscatal.2c01128.

(8) Kilaparthi, S. K.; Addad, A.; Barras, A.; Szunerits, S.; Boukherroub, R. Simultaneous Upcycling of PET Plastic Waste and CO_2 Reduction through Co-Electrolysis: A Novel Approach for Integrating CO_2 Reduction and PET Hydrolysate Oxidation. *J. Mater. Chem. A* **2023**, *11* (47), 26075–26085. https://doi.org/10.1039/D3TA05726G.

(9) Bashir, I.; McGettrick, J. D.; Kühnel, M. F.; Sarfraz, B.; Arshad, S. N.; Rauf, A. Sustainable Formate Synthesis: Integrating Ethylene Glycol Oxidation with Carbon Dioxide Electrocatalysis Using Redox-Stabilized Earth-Abundant Electrodes. *ACS Sustain. Chem. Eng.* **2024**, *12* (12), 4795–4802. https://doi.org/10.1021/acssuschemeng.3c08233.

(10) Liu, H.; Wang, Z.; He, Y.; Hu, X.; Liu, L. Simultaneous Electrochemical Upgrading of Polyethylene Terephthalate Plastic and Carbon Dioxide into Valuable Chemicals. *Appl. Catal. B Environ. Energy* **2025**, *361*, 124667. https://doi.org/10.1016/J.APCATB.2024.124667.

(11) Ma, F.; Li, Z.; Hu, R.; Wang, Z.; Wang, J.; Li, J.; Nie, Y.; Zheng, Z.; Jiang, X. Electrocatalytic Waste-Treating-Waste Strategy for Concurrently Upgrading of Polyethylene Terephthalate Plastic and CO_2 into Value-Added Formic Acid. *ACS Catal.* **2023**, *13* (21), 14163–14172. https://doi.org/10.1021/acscatal.3c03428.

(12) Li, W.; Xiao, D.; Gong, X.; Xu, X.; Ma, F.; Wang, Z.; Wang, P.; Liu, Y.; Dai, Y.; Zheng, Z.; Fan, Y.; Huang, B. Electrocatalytic Upgrading of Polyethylene Terephthalate Plastic to Formic Acid at an Industrial-Scale Current Density via Ni-MOF@MnCo-OH Catalyst. *Chem. Eng. J.* **2024**, *480*,

148087. https://doi.org/10.1016/j.cej.2023.148087.

(13) Lu, X.; Guo, Y.; Fu, H.; Song, J.; Liang, C.; Jiang, H.; Wang, Z.; Liu, Y.; Cheng, H.; Zheng, Z.; Wu, Y.; Wang, P.; Huang, B. Electrochemical Upcycling Strategy for Polyethylene Terephthalate Plastic Coupled with Efficient Hydrogen Production. *Chem. Eng. J.* **2025**, *506*, 159810. https://doi.org/10.1016/j.cej.2025.159810.

(14) Ma, Y.; Ge, H.; Zhang, Y.; Jian, N.; Yu, J.; Arbiol, J.; Li, C.; Zhong, Y.; Li, L.; Kang, H.; Wang, J.; Cabot, A.; Li, J. Selective Electrooxidation of Ethylene Glycol to Formate with Hydrogen Cogeneration in Ni_3S_2 Nanodomains on NiFeMn-LDH Nanosheet Arrays. *ACS Sustain. Chem. Eng.* **2025**, *13* (15), 5601–5612. https://doi.org/10.1021/acssuschemeng.4c10750.

(15) Jiang, Y.; Li, J.; Guo, X.; Chen, Y.; Sun, W.; Peng, C. Electrocatalytic Reforming of Polyethylene Terephthalate Waste Plastics into High-Value-Added Chemicals with Green Hydrogen Generation. *J. Colloid Interface Sci.* **2025**, *685*, 29–37. https://doi.org/10.1016/j.jcis.2025.01.109.

(16) Cao, C.; Ma, D.; Jia, J.; Xu, Q.; Wu, X.; Zhu, Q. Divergent Paths, Same Goal: A Pair-Electrosynthesis Tactic for Cost-Efficient and Exclusive Formate Production by Metal–Organic-Framework-Derived 2D Electrocatalysts. *Adv. Mater.* **2021**, *33* (25), 2008631. https://doi.org/10.1002/adma.202008631.

(17) Wei, X.; Li, Y.; Chen, L.; Shi, J. Formic Acid Electro-Synthesis by Concurrent Cathodic CO_2 Reduction and Anodic CH_3OH Oxidation. *Angew. Chem. Int. Ed.* **2021**, *60* (6), 3148–3155. https://doi.org/10.1002/anie.202012066.

(18) Xiao, C.; Cheng, L.; Wang, Y.; Liu, J.; Chen, R.; Jiang, H.; Li, Y.; Li, C. Low Full-Cell Voltage Driven High-Current-Density Selective Paired Formate Electrosynthesis. *J. Mater. Chem. A* **2022**, *10* (3), 1329–1335. https://doi.org/10.1039/D1TA08303A.

(19) Bajada, M. A.; Roy, S.; Warnan, J.; Abdiaziz, K.; Wagner, A.; Roessler, M. M.; Reisner, E. A Precious-Metal-Free Hybrid Electrolyzer for Alcohol Oxidation Coupled to CO_2 -to-Syngas Conversion. *Angew. Chem. Int. Ed.* **2020**, *59* (36), 15633–15641. https://doi.org/10.1002/anie.202002680.

(20) Wang, Y.; Gonell, S.; Mathiyazhagan, U. R.; Liu, Y.; Wang, D.; Miller, A. J. M.; Meyer, T. J. Simultaneous Electrosynthesis of Syngas and an Aldehyde from CO_2 and an Alcohol by Molecular Electrocatalysis. *ACS Appl. Energy Mater.* **2019**, *2* (1), 97–101. https://doi.org/10.1021/acsaem.8b01616.
